# Different effects of constitutive and induced microbiota modulation on microglia in a mouse model of Alzheimer’s disease

**DOI:** 10.1186/s40478-020-00988-5

**Published:** 2020-07-29

**Authors:** Charlotte Mezö, Nikolaos Dokalis, Omar Mossad, Ori Staszewski, Jana Neuber, Bahtiyar Yilmaz, Daniel Schnepf, Mercedes Gomez de Agüero, Stephanie C. Ganal-Vonarburg, Andrew J. Macpherson, Melanie Meyer-Luehmann, Peter Staeheli, Thomas Blank, Marco Prinz, Daniel Erny

**Affiliations:** 1 Institute of Neuropathology, University of Freiburg, Breisacher Str. 64, 79106 Freiburg, Germany; 2 Faculty of Biology, University of Freiburg, Freiburg, Germany; 3 Maurice E. Müller Laboratories, Department for Biomedical Research (DBMR), University Clinic of Visceral Surgery and Medicine, Inselspital, University of Bern, Bern, Switzerland; 4 Institute of Virology, Medical Center University of Freiburg, Freiburg, Germany; 5 Department of Neurology, Medical Center University of Freiburg, Freiburg, Germany; 6 Center for Basics in NeuroModulation (NeuroModulBasics), Faculty of Medicine, University of Freiburg, Freiburg, Germany; 7 Signalling Research Centres BIOSS and CIBSS, University of Freiburg, Freiburg, Germany; 8 Berta-Ottenstein-Programme, Faculty of Medicine, University of Freiburg, Freiburg, Germany

**Keywords:** Gut microbiota, Antibiotics, Germ-free, Microglia, Alzheimer’s disease

## Abstract

It was recently revealed that gut microbiota promote amyloid-beta (Aβ) burden in mouse models of Alzheimer’s disease (AD). However, the underlying mechanisms when using either germ-free (GF) housing conditions or treatments with antibiotics (ABX) remained unknown. In this study, we show that GF and ABX-treated 5x familial AD (5xFAD) mice developed attenuated hippocampal Aβ pathology and associated neuronal loss, and thereby delayed disease-related memory deficits. While Aβ production remained unaffected in both GF and ABX-treated 5xFAD mice, we noticed in GF 5xFAD mice enhanced microglial Aβ uptake at early stages of the disease compared to ABX-treated 5xFAD mice. Furthermore, RNA-sequencing of hippocampal microglia from SPF, GF and ABX-treated 5xFAD mice revealed distinct microbiota-dependent gene expression profiles associated with phagocytosis and altered microglial activation states. Taken together, we observed that constitutive or induced microbiota modulation in 5xFAD mice differentially controls microglial Aβ clearance mechanisms preventing neurodegeneration and cognitive deficits.

## Introduction

Alzheimer’s disease (AD) is the most common neurodegenerative disease and cause of dementia worldwide [9]. The progressive loss of neurons causes symptoms such as memory loss and cognitive decline. Despite enormous efforts in AD research, the etiology of the frequent sporadic form of AD remains largely unknown [47]. The hereditary familial form of AD accounts only for less than 5% of all cases of AD and typically has a much earlier onset [51]. The main hallmarks of this incurable disease in the brain are extracellular amyloid-beta (Aβ) depositions and intracellular neurofibrillary tangles. The early aggregation and cerebral deposition of Aβ is suggested to take place decades before the first symptoms appear [48], and Aβ depositions mainly drive the development and progression of AD [53]. In the central nervous system (CNS) microglia represent the main innate immune cells, and in contrast to other tissue macrophages have a unique solely yolk-sac origin [17, 24, 46]. While they are essential for maintaining tissue homeostasis during physiological conditions [25], microglia continuously survey their microenvironment, react to pathological stimuli and are involved in virtually all CNS diseases including AD pathogenesis [39, 44]. Recent genome-wide association studies (GWAS) showed that genes mainly or exclusively expressed by microglia, including complement receptors, or triggering receptor expressed on myeloid cells-2 (*TREM2*) are associated with increased risk of developing AD [28]. Microglia sense Aβ by several receptors (i.e. TREM2) resulting in microglial activation, accumulation around Aβ plaques and production of potential neurotoxic proinflammatory cytokines [44, 59]. As the professional phagocytes of the CNS, microglia play a crucial role in the removal of Aβ aggregates [44]. Interestingly, microglia in *Trem2-deficient* 5xFAD mice incorporated less Aβ than microglia from *Trem2*-expressing 5xFAD mice [60].

Growing body of evidence highlight a considerable influence of gut microbes on the host’s health [21]. Microbiota-derived molecules, such as short-chain fatty acids (SCFAs), have been shown to contribute to host’s physiology and boost immune functions [21]. We have previously demonstrated that the maturation and function of microglia were highly affected by host gut microbes [12]. In mice born and raised under germ-free (GF) conditions, microglia displayed an immature homeostatic phenotype defined by altered gene expression, increased numbers, and hyper-ramified morphology. In addition, they displayed compromised immune response upon acute challenge with bacterial molecules and virus infection [12]. Notably, temporal eradication of gut bacteria by antibiotic treatment (ABX) in former colonized adult SPF mice induced a comparable microglial phenotype to GF mice, arguing that constant signals from gut bacteria are necessary for proper microglial homeostasis und function [12]. Remarkably, the pathophysiology of several CNS diseases including AD was recently linked to host microbiota [14]. Harach and colleagues uncovered that AD pathology is diminished in the double transgenic APPPS1 AD mouse model under GF housing conditions compared to colonized controls [18]. Furthermore, postnatal long-term microbiota manipulation by ABX reduced Aβ burden in APP_SWE_/PS1_ΔE9_ mice compared to non-treated controls [34]. However, the precise role of microglia, the involved cellular mechanism, the impact on cognitive function, and whether the production or degradation of Aβ is affected, remained unclear.

In order to gain mechanistic insights, we characterized the Aβ-associated pathology and related hippocampus-associated behavior at an early and progressed stage of the disease using 5x familial AD (5xFAD) mice bred under both GF and specific pathogen free (SPF) conditions and induced gut bacteria depletion by ABX in a third cohort. Additionally, we thoroughly investigated the influence of the constant and induced depletion of gut bacteria on microglia-mediated effects during Aβ pathology. We revealed that constitutive absence of gut microbiota in GF 5xFAD mice increased the microglial uptake of Aβ deposits in the hippocampus, resulting in decreased Aβ burden, associated neuronal loss and retained hippocampus-associated memory function, without affecting the production of Aβ. While ABX-induced acute depletion of gut bacteria resulted in similar decrease of Aβ depositions, this effect was not attributed to microglial Aβ phagocytosis.

## Materials and methods

### Mice

As a mouse model of Alzheimer’s disease we used heterozygous male 5xFAD transgenic mice and non-transgenic littermates (4 and 10 months of age) on a C57BL/6 J background co-expressing human APP^K670N/M671L (Sw) + I716V (Fl) + V717I(Lo)^ and PS1^M146L + L286V^ under the control of the neuron-specific Thy-1 promoter [38]. Mice were housed under specific pathogen-free (SPF) conditions under a 12-h light, 12-h dark cycle with food and water ad libitum at CEMT (Freiburg, Germany). GF 5xFAD mice were generated via embryo transfer by Kathleen McCoy. GF heterozygous 5xFAD and WT littermates were obtained from the Clean Mouse Facility (Bern, Switzerland). In order to deplete microbiota, mice were treated orally via drinking water with a mixture of antibiotics (ABX), containing 1 mg/ml vancomycin (Hexal), 1 mg/ml cefoxitin (Santa Cruz Biotechnology), 1 mg/ml gentamicin (Sigma-Aldrich) and 1 mg/ml metronidazol (Sigma-Aldrich) for 2 months as described previously [12]. All animal experiments were approved by the Ministry for Nature, Environment and Consumers` Protection of the state of Baden-Württemberg and were performed in accordance to the respective national, federal, and institutional regulations (G19–02 and X16-04A).

### Histology & Immunofluorescence

Mice were deeply anesthetized by intraperitoneal injection of a mixture of ketamine (100 mg/kg body weight) and xylazine (10 mg per kg body weight) and transcardially perfused with ice-cold 1xPBS. Collected brains were post-fixed in 4% PFA for 24 h. One hemisphere was cryopreserved in 30% sucrose for 48 h, whereas the other hemisphere was paraffin embedded. Cryopreserved brains were frozen on dry ice and cut into 25 μm thick coronal serial sections on a sliding microtome (SM2000R, Leica Biosystems) and collected in 5% glycerol. As described previously [66], every tenth brain sections containing hippocampus from rostral to caudal were immunolabeled free-floating by incubating with anti-Iba1 antibody (rabbit, 1:500, Wako) and anti-Clec7a (1:30, Invivo Gen) for 24 h at 4 °C. Subsequently, sections were incubated with Alexa-Fluor-488 and 647-conjugated secondary antibody (1:500, Life technologies) for 24 h at 4 °C. Compact Aβ plaques were stained with 2 μM thiazine red (TR) (Sigma-Aldrich), a fluorescent congo red derivate, for 5 min at room temperature (RT). Finally, nuclei were stained with DAPI (4′,6-diamidin-2-phenylindol, 1:10000) for 10 min at RT. Additional series of brain sections were immunolabeled to visualize compact Aβ-plaques, as well as 1–16 Aβ precursor forms by using anti-6E10 antibody (1:1000, Biolegend) and anti-Iba1 antibody (1:500, Wako) at 4 °C for 24 h, followed by incubation with corresponding conjugated secondary antibodies Alexa-Fluor-568 and 488 (1:500, Life technologies) for 24 h at 4 °C. DAPI was used as a nuclear stain for 10 min at RT. Aβ-plaque load and density of Iba1-immunoreactive cells were quantified throughout the entire hippocampus. In total 10 to 12 sections were analyzed per mouse.

Paraffin-embedded brains were cut sagittal into 3 μm thin sections and immunofluorescence labeling was performed by using following primary antibodies: anti-NeuN (1:200, Abcam), anti-Iba1 (1:500, Synaptic System), anti-P2ry12 (1:200, AnaSpec) and anti-ApoE (1:50, Merck), upon antigen retrieval (pH 6.0 citrate) at 4 °C for 24 h. Subsequently, respective conjugated secondary antibodies Alexa-Fluor 647 or 488 (1:500, Life technologies) were used for 2 h at RT. Aβ plaques were visualized by TR for 5 min at RT. Nuclei were stained with DAPI (1:10000) for 10 min at RT. Fluorescence images were taken with BZ-9000 Biorevo microscope (Keyence). Sections were analyzed by using BZ-II Analyzer Software (Keyence). Confocal images were taken with Olympus Fluoview FV 1000 confocal laser scanning microscope (Olympus).

### Behavior analysis

Spatial working memory was tested using the continuous spontaneous alternation task in a T-maze as described previously [10, 49]. In short, animals were set into the base of a T-maze and forced to explore one of the T-maze arms until it returned to the base arm. Subsequently, the blocked arm was opened, and the animal could explore the maze. Once one arm of the T-maze was entered, the other arm was blocked until the animal returned to the base arm. Then, the exit of the base arm was blocked for 5 s and the animal explored the maze again. The experiment was stopped after 14 free choice arm entries. Arm entries were scored as alternations if an animal chose the opposing arm compared to the arm visited immediately prior to the scored instance. Repetitive arm entries were scored as entering the same arm for the third or more consecutive time. The total time after 14 free choice arm entries was recorded.

The Novel Object Recognition (NOR) task was applied to evaluate recognition memory as published previously [2, 37]. During the habituation phase, each mouse could explore two similar objects within a total exploration time of 20 s. We commenced the testing phase 6 h after the habituation phase. During the testing session, each mouse could explore a familiar object and a novel object of different shape and texture. The position of the novel object and the familiar object was randomized between each mouse. The time spent by each mouse to explore the novel object and the familiar object was noted. The experiment was stopped when the total exploration time reached 20 s. The time spent by each mouse to explore the novel object and the familiar object, as well as the total time for paradigm completion during the testing phase was recorded.

### Ex vivo microglia isolation and flow cytometry

Hippocampi were collected and microglia were enriched by using density gradient separation and were prepared as described previously [12, 65]. The cell suspension was then incubated with Fc receptor blocking antibody CD16/CD32 (1:200, BD Bioscience) and Fixable Viability Dye eFluor® 780 (1:1000, eBioscience) for 10 min at 4 °C. Subsequently, the following antibodies were used: anti-CD11b (1:200, clone M1/70, Biolegend), anti-CD45 (1:200, clone 30-F11, BioLegend), anti-CD11c (1:100, clone N418, Biolengend) and for lineage exclusion by a dump gate anti-CD3 (1:300, clone 17A2, Biolegend), anti-CD19 (1:300, clone 6D5, Biolegend), anti-CD45R (1:300, clone RA3-6B2, BD Bioscience), anti-Ly6C (1:300, clone AL-21, BD Bioscience) and anti-Ly6G (1:300, clone 1A8, BD Bioscience) and incubated for 30 min at 4 °C.

### Ex vivo Aβ phagocytosis assay

Mice were injected intraperitoneally with methoxy-X-O4 (Tocris) (10 mg/kg bodyweight), a fluorescent congo red derivate, in a DMSO/PBS mixture. After 3 h, hippocampi were collected and microglial cells were assessed as described previously [65]. Percentage of methoxy-XO-4 positive microglia were determined by flow cytometry using a FACS Canto II (BD Bioscience) and analyzed with FlowJo software (Tree Star).

### Microbial profiling of caecal contents

DNA extraction, 16S rRNA sequencing and computation analysis of caecal contents from 4 months old SPF and ABX-treated 5xFAD mice and respective WT controls was performed as described previously [62] with slight modifications. 12 SPF and 16 ABX-treated 5xFAD mice, as well as 15 SPF and 14 ABX-treated WT mice were analyzed. In brief, caecal contents were collected in 2 ml microfuge tubes and stored at − 80 °C prior to DNA extraction. Total DNA was isolated from samples using the QIAamp DNA stool kit (Qiagen) according to the modified manufacturer’s instructions. Afterwards, 100 - 400 ng of DNA samples were subjected to amplification of V5/V6 region of bacterial 16S rRNA. Bacteria-specific primers (forward 5′ CCATCTCATCCCTGCGTGTCTCCGACTCAGC barcode ATTAGATACCCYGGTAGTCC 3′ and reverse 5′ CCTCTCTATGGGCAGTCGGTGATACGAGCTGACGACARCCATG-3′) were used. Amplicon sequencing was performed using the Ion PGM™ Sequencing 400 Kit and Ion 316™ Chip V2 within the Ion PGM™ System (Thermo Fisher). Fastq sequencing files were first loaded into the QIIME 1.9.1 pipeline [8], using custom analysis scripts for analysis on the UBELIX Linux cluster of the University of Bern [62]. The *biom* file and mapping file were used for statistical analyses and data visualization in the R with package *phyloseq*. The α-diversity (Observed OTUs, Simpson and Shannon index), β-diversity (Bray-Curtis genus-level community dissimilarities), and statistical analysis of clustering using Mann-Whitney U tests for alpha diversity and Adonis (PERMANOVA) for beta diversity to confirm that the strength and statistical significance of groups in the same distance metrics in *phyloseq* using R [33]. The multivariate analysis by linear models (MaAsLin) R package were used to find associations between metadata and microbial community abundance [36]. Plots were generated with ggplot2 using *phyloseq* object.

### Quantification of bacterial load by flow cytometry

Fecal samples were collected from SPF, GF and ABX-treated mice and weighted, immediately homogenized in ice-cold 1xPBS and filtered through 50 μm CellTrics filters (Sysmex). A fraction of the filtrates was diluted 1:20 in 1xPBS and centrifuged for 5 min at 3000 g at 4 °C. Subsequently, supernatant was aspirated and Syto9 (1:1000 in PBS, Thermo Fisher), a dye to identify gram+ and gram- bacteria was added for 10 min at 4 °C. DAPI (1:1000) was used for dead cell exclusion and the percentage of live bacteria was recorded by using flow cytometry. Flow cytometry cell counting beads (1:20, Thermo Fisher) were added to quantify absolute number of live bacteria per mg fecal sample.

### RNA-sequencing

Total RNA was extracted from FACS sorted viable CD11b^+^CD45^low^DUMP^−^ hippocampal microglia cells using the ARCTURUS® *PicoPure*® *RNA* Isolation Kit (Thermo Fisher) according to manufacturer’s protocol. The SMARTer Ultra Low Input RNA Kit for Sequencing v4 (Clontech Laboratories, Inc., Mountain View, CA, USA) was used to generate first strand cDNA from 300 pg total-RNA. Double stranded cDNA was amplified by LD PCR (13 cycles) and purified via magnetic bead clean-up. Library preparation was carried out as described in the Illumina Nextera XT Sample Preparation Guide (Illumina, Inc., San Diego, CA, USA). 150 pg of input cDNA were tagmented (tagged and fragmented) by the Nextera XT transposome. The products were purified and amplified via a limited-cycle PCR program to generate multiplexed sequencing libraries. For the PCR step 1:5 dilutions of index 1 (i7) and index 2 (i5) primers were used. The libraries were quantified using the KAPA SYBR FAST ABI Prism Library Quantification Kit (Kapa Biosystems, Inc., Woburn, MA, USA). Equimolar amounts of each library were pooled, and the pools were used for cluster generation on the cBot with the Illumina TruSeq SR Cluster Kit v3. The sequencing run was performed on a HiSeq 1000 instrument controlled by the HiSeq Control Software (HCS) 2.2.38, using the indexed, 50 cycles single-read (SR) protocol and the TruSeq SBS v3 Reagents according to the Illumina HiSeq 1000 System User Guide. Image analysis and base calling were done by the Real Time Analysis Software (RTA) 1.18.61. The resulting .bcl files were converted into fastq files with the CASAVA Software 1.8.2. Library preparation and RNAseq were performed at the Genomics Core Facility “KFB - Center of Excellence for Fluorescent Bioanalytics” (University of Regensburg, Regensburg, Germany; www.kfb-regensburg.de).

Fastq files were quality controlled using FastQC [1] and reads were mapped to the GRCm38 mouse genome using the Star aligner [11]. Read counts were obtained using the featureCounts program [30] in conjunction with the Gencode transcriptome version M21 [15]. Differential gene expression analysis was performed using the limma/voomWithQualityWeights pipeline in R [29, 31]. Venn diagram was generated by using previously published tools [19]. Heatmaps were generated using the package pheatmap [40]. Pathway analysis was performed using Ingenuity Pathway Analysis (IPA, QIAGEN).

### Elisa

Quantification of soluble and insoluble Aβ40 and 42 species in hippocampal homogenates were quantified by performing enzyme-linked immunosorbent assay as described previously [66]. Briefly, hippocampi were harvested and homogenized (10% w/v) in 1xPBS containing protease inhibitor and sequentially extracted with PBS (soluble fraction), PBS + 0.1% Triton X-100 (membrane bound fraction) and finally with 8 M guanidine hydrochloride solution (insoluble fraction). Protein concentration in each fraction was measured by using Bradford assay (Carl Roth) and ELISA was performed using Human Aβ42 ultrasensitive ELISA kit and Human Aβ40/Aβ42 ELISA kits (Invitrogen) according to manufacturer’s protocols.

### Western blot analysis

Hippocampi were harvested and homogenized in RIPA buffer (25 mM Tris-HCl, 150 mM NaCl, 1% Nonidet P-40, 0.5% sodium deoxycholate, 0.1% SDS, protease inhibitor, pH 7.5) to extract total protein. Total protein concentration was determined by using Bradford assay (Carl Roth). Samples were separated by 4–12% NuPAGE Bis-tris mini gels (Invitrogen) and immunoblotted using antibodies against APP and CTFs (1:3000, Sigma), BACE1, ADAM10 (1:1000, Cell Signaling Technology), Nicastrin, PEN2, Presenilin 1 and 2 (PS1, PS2) (1:1000, γ Secretase Antibody Sampler Kit, Cell Signaling Technology) and anti-Aβ (6E10, 1:3000, Biolegend) for 24 h at 4 °C. Anti-β-actin-HRP (1:5000, Abcam) was used as loading control. Immunoblots were incubated with corresponding HRP-linked secondary antibodies for 1 h at RT and visualized by using SuperSignal™ West Femto Maximum Sensitivity Substrate (Thermo Fisher).

### Statistical analysis

Statistical analysis was performed using GraphPad Prism (GraphPad Software, Version 5.0, La Jolla, USA). All data were tested for normality applying the Shapiro-Wilk normality test. If normality was given, either an unpaired *t* test, one-way ANOVA followed by Tukey’s *post-hoc* comparison test or two-way ANOVA followed by Bonferroni’s compression was applied respectively. Differences were considered significant when *P* value < 0.05. To obtain unbiased data, experimental mice were all processed together. Cell quantifications were performed blinded by two scientists independently and separately.

## Results

### Absence of gut microbiota ameliorates hippocampal Aβ burden of 5xFAD mice

To investigate whether gut microbiota is detrimental or beneficial during neurodegeneration, we took advantage of the 5xFAD mouse model that recapitulates major features of the Aβ pathology [38]. In 5xFAD mice, Aβ plaques start to appear at around 2 months of age [38]. In order to evaluate the modulation of an early phase of the disease in 5xFAD mice by gut microbiota, we first compared 4 months old GF 5xFAD to colonized (specific pathogen free, SPF) 5xFAD mice. Additionally, we treated SPF 5xFAD mice orally with ABX for 2 months prior to analyze at an age of 4 months.

**Fig. 1:**
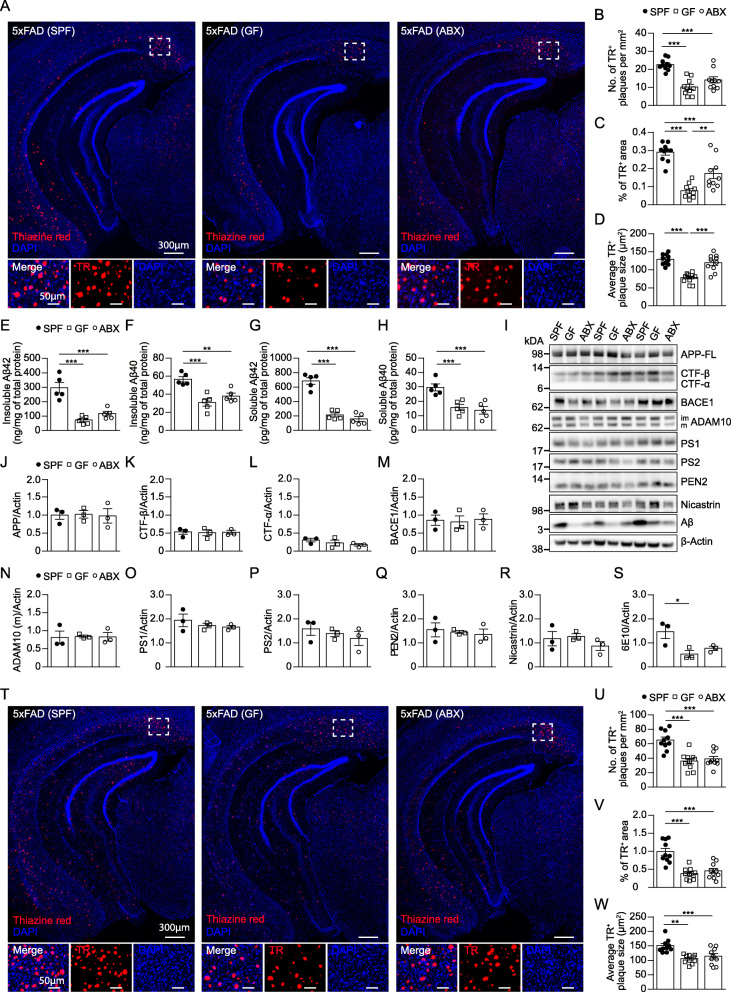
Absence of host microbiota reduces hippocampal Aβ depositions of 5xFAD mice. (**a**) Representative fluorescence images of thiazine red^+^ (TR; red) compact Aβ plaques in the hippocampus of 4 months old SPF, GF and ABX-treated 5xFAD mice**.** Nuclei were stained with DAPI (blue). Overview of hippocampus and magnification of subiculum (dashed line) are shown. Scale bars represent 300 μm (overview) and 50 μm (insert). (**b**) Quantification of the number of TR^+^ Aβ-plaques per mm^2^, (**c**) percentage of TR^+^ area and (**d**) average TR^+^ plaque size (μm^2^) in coronal hippocampal sections. Each symbol represents one mouse. Data are presented as mean ± s.e.m. Significant differences were determined by one-way ANOVA followed by Tukey’s post-hoc comparison test (*P < 0.05, **P < 0.01, ***P < 0.001). Data are representative of four independent experiments. Enzyme-linked immunosorbent assay (ELISA) for **(e)** insoluble Aβ42, **(f)** insoluble Aβ40, **(g)** soluble Aβ42 and **(h)** soluble Aβ40 fractions of hippocampal brain extracts from 4 months old SPF, GF and ABX-treated 5xFAD mice. Each symbol represents one mouse. Data are presented as mean ± s.e.m. Significant differences were determined by one-way ANOVA followed by Tukey’s post-hoc comparison test (**P < 0.01, ***P < 0.001). Data are representative of two independent experiments. (**i**) Representative immunoblots of hippocampal brain homogenates from 4 months old SPF, GF and ABX-treated 5xFAD mice against human full-length amyloid precursor protein (APP-FL), C-terminal fragment (CTF) α, CTF-β, β-site of APP cleaving enzyme (BACE) 1, A Disintegrin And Metalloproteinase (ADAM10), γ-secretase complex (Nicastrin, presenilin enhancer (PEN) 2, Presenilin (PS) 1, PS2) and Aβ (6E10). β-Actin was used as loading control. Each lane represents one mouse. Quantification of (**j**) APP-FL, (**k**), CTF-β, (**l**) CTF-α, (**m**) BACE1, (**n**) ADAM10, (**o**) PS1, (**p**) PS2, (**q**) PEN2, (**r**) Nicastrin, and (**s**) Aβ protein levels normalized to β-Actin are shown. Each symbol represents one mouse. Data are presented as mean ± s.e.m. Significant differences were determined by one-way ANOVA followed by Tukey’s post-hoc comparison test (*P < 0.05). Data are representative of two independent experiments. (**t**) Representative fluorescence images of TR^+^ compact Aβ plaques in the hippocampus of 10 months old 5xFAD mice. Nuclei were stained with DAPI (blue). Overview of hippocampus and magnification of subiculum (dashed line) are shown. Scale bars represent 300 μm (overview) and 50 μm (insert). (**u**) Quantification of the number of TR^+^ Aβ-plaques per area (mm^2^), (**v**) percentage of TR^+^ area and (**w**) average of TR^+^ plaque size (μm^2^). Each symbol represents one mouse. Data are presented as mean ± s.e.m. Significant differences were determined by one-way ANOVA followed by Tukey’s post-hoc comparison test (**P < 0.01, ***P < 0.001). Data are representative of four independent experiments

GF-housed and ABX-treated mice displayed enlarged caeca with dark-colored caecal contents and incremented caecal weights compared to SPF controls, as previously described [12] (Suppl. Fig. 1A-C). To confirm the successful reduction of gut bacteria after ABX application, we quantified the number of live bacteria in fecal samples by flow cytometry (Suppl. Fig. 1D - F). In line, we observed a significant reduction in species richness in ABX-treated 5xFAD mice and wild-type (WT) littermates (Suppl. Fig. 1G & H). In contrast to a recent report [5], we did not detect substantial differences in species richness (q-value < 0.05) in caecal contents of SPF 5xFAD and non-transgenic littermates (Suppl. Fig. 1I - K and Additional files 6 and 7). First, to analyze the amount of Aβ depositions by histopathological assessment in hippocampi of 4 months old SPF, GF and ABX-treated 5xFAD mice, we took advantage of the fluorescent congo red derivate thiazine red (TR) which visualizes compact Aβ depositions (Fig. 1A). In line with previous observations in other AD mouse models [18, 34], the amount of TR^+^ compact plaques was diminished in hippocampi of GF and ABX-treated 5xFAD mice compared to SPF 5xFAD controls (Fig. 1A - C). Additional immunofluorescence labelling with the 6E10 antibody, targeting compact and diffuse Aβ, showed similar results (Suppl. Fig. 2A - C). Of note, we observed significantly smaller plaques under GF conditions compared to SPF and ABX-treated 5xFAD mice (Fig. 1D & Suppl. Fig. 2D). In accordance, both insoluble Aβ42 and Aβ40 (Fig. 1E & F, Suppl. Fig. 2E) as well as soluble Aβ42 and Aβ40 fractions (Fig. 1G & H, Suppl. Fig. 2F) were significantly decreased in hippocampal brain homogenates of GF and ABX 5xFAD mice when compared to SPF 5xFAD controls. To assess a potential effect of gut microbiota on the processing of the amyloid precursor protein (APP), we measured APP and its C-terminal fragments (CTF-α, CTF-β) as well as secretases involved in APP-processing, including β-secretase (β-site of APP cleaving enzyme, ΒΑCΕ1), A Disintegrin And Metalloproteinase (ADAM)10 and components of the γ-secretase complex (Nicastrin, PEN2, Presenilin1, Presenilin 2) in hippocampal tissue extracts by immunoblotting and detected no alterations (Fig. 1I – R). Consistent with the ELISA results, western blot analysis also demonstrated significantly lower Aβ levels in both GF and ABX 5xFAD mice compared to SPF 5xFAD animals (Fig. 1S).

Next, we asked whether the Αβ accumulation was modulated at a later progressed stage of AD pathology by either constant or induced microbial absence. Therefore, we investigated SPF and GF housed 5xFAD mice at an age of 10 months. Additionally, ABX treatment of SPF 5xFAD mice was started 2 months prior to analysis at an age of 10 months. Compared to the 4 months old SPF 5xFAD mice, we noticed a robust increase of TR^+^ Aβ depositions in 10 months old SPF 5xFAD animals, whereas GF and ABX-treated 5xFAD mice developed significantly less and smaller Aβ plaques (Fig. 1T - W). Consequently, both insoluble and soluble Aβ42 and Aβ40 fractions were decreased in hippocampi of GF and ABX 5xFAD mice compared to SPF 5xFAD controls (Suppl. Fig. 2G - K). APP processing also showed no significant alterations in the aged GF 5xFAD mice (Suppl. Fig. 2L – U). Altered Aβ load was additionally detected by immunoblot and 6E10 immunofluorescence labelling (Suppl. Fig. 2V – Z), indicating a possible modulation of Aβ pathology even at later stages of the disease. In sum, our findings highlight a disease-promoting role of gut bacteria during Aβ-mediated neurodegeneration, whereas modulation thereof might improve the disease course.

### Gut bacteria influence memory function of 5xFAD mice

**Fig. 2:**
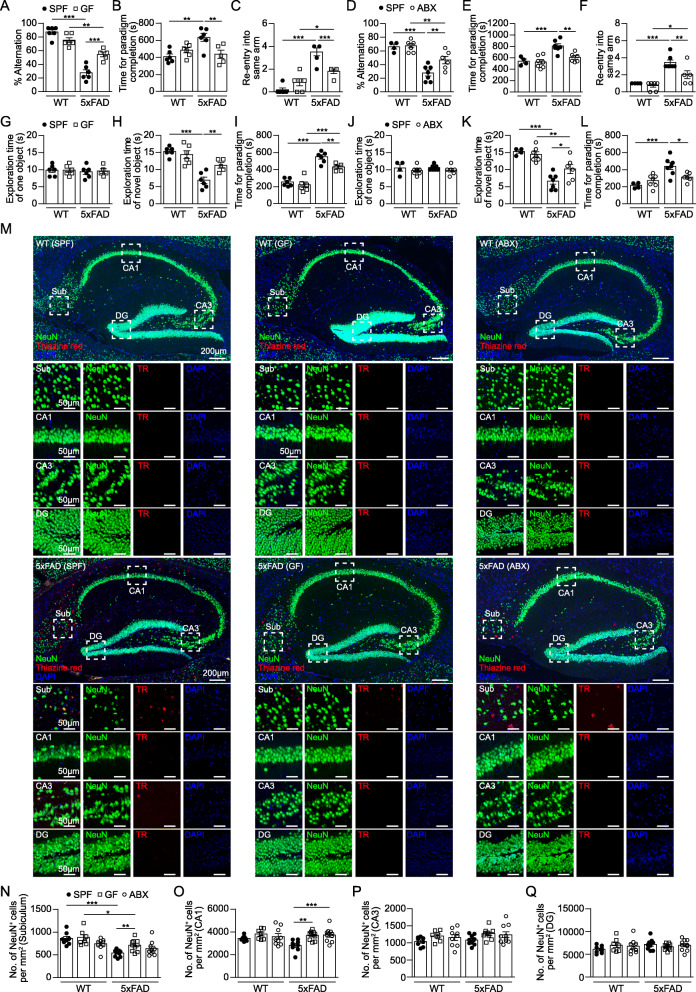
Restored memory deficits in 5xFAD mice lacking microbes. (**a-c**) T-maze test performance of 10 months old SPF and GF 5xFAD mice, as well as aged-matched WT controls or (**d-f**) of 10 months old SPF and ABX 5xFAD mice, as well as WT controls. (**g-i**) Novel object recognition test (NOR) of 10 months old SPF and GF 5xFAD mice, as well as age-matched WT controls or (**j-l**) of 10 months old SPF and ABX 5xFAD mice, as well as respective WT controls. Each symbol represents one mouse. Data are presented as mean ± s.e.m. Significant differences were determined by two-way ANOVA followed by Bonferroni’s post-hoc comparison test (*P < 0.05, **P < 0.01, ***P < 0.001). Data are representative of three independent experiments. (**m**) Representative immunofluorescence images of NeuN^+^ neurons (green) and TR^+^ (red) compact Aβ plaques in the subiculum (Sub), cornu ammonis (CA) 1, CA3 and dentate gyrus (DG) of the hippocampus of 10 months old SPF, GF and ABX-treated 5xFAD mice**.** Nuclei were stained with DAPI (blue). Overview of hippocampus and magnifications of subiculum, CA1, CA3 and DG (dashed lines) are shown. Scale bars represent 200 μm (overview) and 50 μm (inserts). Quantification of the number of NeuN^+^ neurons per mm^2^ in the subiculum (**n**), CA1 (**o**), CA3 (**p**) and DG (**q**) of sagittal hippocampal sections from SPF, GF and ABX-treated 5xFAD and age-matched WT mice. Each symbol represents one mouse. Data are presented as mean ± s.e.m. Significant differences were determined by two-way ANOVA followed by Bonferroni’s post-hoc comparison test (**P < 0.01, ***P < 0.001). Data are representative of two independent experiments

To elucidate whether the reduced Aβ plaque burden in 10 months old GF and ABX 5xFAD mice compared to the steadily colonized SPF 5xFAD controls is affecting cognitive function, we first examined spatial working memory in the T-maze paradigm. As expected, SPF 5xFAD mice showed decreased percentage of arm alternation and more re-entries into the same arm of the T-maze (Fig. 2A - C) compared to age-matched SPF WT littermates. Both GF housing (Fig. 2A – C & Suppl. Fig. 3A) and oral ABX application (Fig. 2D – F & Suppl. Fig. 3B) partially rescued this phenotype in 5xFAD mice, whereas the SPF, GF and ABX WT controls showed no behavioral abnormalities. Furthermore, when tested in the novel object recognition (NOR) task, GF 5xFAD (Fig. 2G - I) as well as ABX-treated 5xFAD mice (Fig. 2J - L) spent significantly more time exploring the novel object compared to SPF 5xFAD mice, suggesting partially protected learning and recognition memory. Importantly, impairment of spatial and recognition memory in colonized SPF 5xFAD mice was accompanied by prominent neuronal loss in the subiculum (sub) as well as in the cornu ammonis (CA) 1 region, while we did not observe significant neuronal loss in the CA3 and dentate gyrus (DG) (Fig. 2M – Q). Microbiota manipulation at early disease stages in 4 months old 5xFAD mice did not yet affect markedly hippocampus-associated memory functions (Suppl. Fig. 3C - P) or neuronal survival (Suppl. Fig. 4Q & R). Collectively, our data show a detrimental role of microbiota in the 5xFAD mouse model worsening spatial and recognition memory along with reduced neuronal numbers in the hippocampus.

### Constitutive lack of microbiota augments microglial uptake of Aβ debris

**Fig. 3:**
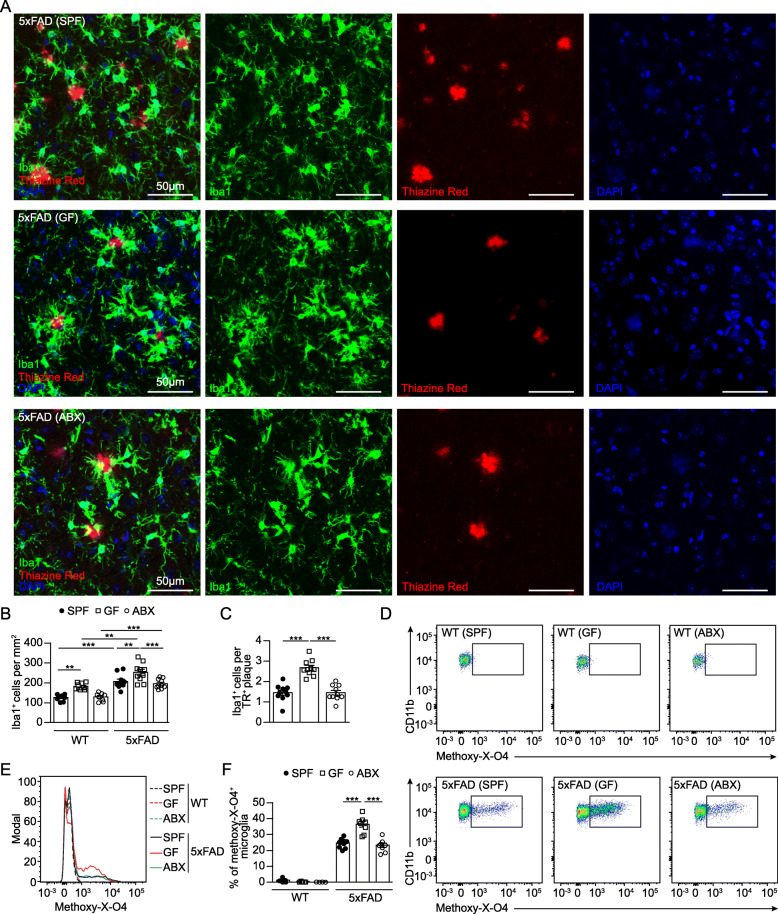
Increased microglial phagocytosis in the hippocampus of 4 months old GF 5xFAD mice. (**a**) Representative immunofluorescence images of TR^+^ (red) Aβ depositions and Iba1^+^ (green) microglia on coronal hippocampal sections of SPF, GF and ABX-treated 5xFAD mice and age-matched WT controls. Nuclei were stained with DAPI (blue). Scale bar: 50 μm. Quantification of (**b**) Iba1^+^ parenchymal microglia in hippocampus of 5xFAD and age-matched WT mice. Quantification of (**c**) TR^+^ plaque-associated microglia in 5xFAD mice. Each symbol represents one mouse. Data are presented as mean ± s.e.m. Significant differences were determined by two-way ANOVA followed by Bonferroni’s post-hoc comparison test or by one-way ANOVA followed by Tukey’s post-hoc comparison test (**P < 0.01, ***P < 0.001). Data are representative of four independent experiments. (**d**) Gating of CD11b^+^methoxy-XO-4^+^ microglia from SPF, GF and ABX-treated 5xFAD mice and age-matched WT controls. Representative dot plots are shown. (**e**) Representative cytometric graph of methoxy-X-O4^+^ labelled microglia from SPF (black line), GF (red line) and ABX-treated (blue line) 5xFAD mice and respective WT controls (dashed lines). (**f**) Quantification of percentages of methoxy-X-O4^+^ labelled microglia cells are depicted. Each symbol represents one mouse. Data are presented as mean ± s.e.m. Significant differences were determined by two-way ANOVA followed by Bonferroni’s post-hoc comparison test (***P < 0.001). Data are representative of four independent experiments

We previously demonstrated that microglial features are essentially steered by the host microbiota [12]. In addition, it is widely accepted that microglia critically contribute to AD progression [44]. Following our own and previous observations [18, 34] showing that microbiota have a negative impact on AD pathology in different mouse models of AD, we aimed to clarify the cellular mechanism resulting in decreased Aβ pathology under GF or ABX conditions. To examine the functional role of microglia in 4 months old SPF, GF and ABX-treated 5xFAD mice, we first quantified Iba1^+^ parenchymal microglia numbers in the hippocampi and compared them to the respective WT controls (Fig. 3A & B**,** Suppl. Fig. 4A). 5xFAD mice housed under GF conditions displayed an higher microglial density in the hippocampal tissue compared to SPF 5xFAD mice, while ABX treatment did neither affect microglia density in 5xFAD mice nor WT mice in line with previous findings [12, 54] (Fig. 3A & B**,** Suppl. Fig. 4A). Next, we examined in detail microglia located in a close proximity (< 10 μm) to TR^+^ Aβ plaques. We noticed significantly more TR^+^ Aβ plaque-associated Iba1^+^ microglia in GF 5xFAD mice (Fig. 3A & C), whereas SPF and ABX-treated 5xFAD mice showed less accumulation of Iba1^+^ microglia around TR^+^ Aβ plaques. Similar results were found by using 6E10 immunofluorescence for compact and diffuse Aβ debris (Suppl. Fig. 4B & C). It has been suggested that plaque-associated microglia are critical in preventing senile Aβ plaque formation in AD mouse models, for example by increased phagocytosis [23, 27]. Hence, we investigated whether permanent absence or induced microbiota depletion alters microglial phagocytosis by performing ex vivo flow cytometric based analysis of Aβ uptake using methoxy-X-O4 staining as described before [65] (Fig. 3D & E**,** Suppl. Fig. 5A). In line with the increased Aβ plaque-associated Iba1^+^ microglia numbers in GF 5xFAD mice, we observed higher percentage of methoxy-X-O4^+^ microglial cells under GF conditions (Fig. 3D - F), indicating higher Aβ uptake. Notably, 2 months supplementation of ABX via drinking water did not affect the percentage of methoxy-X-O4^+^ microglial cells which was comparable to SPF levels (Fig. 3D – F). These findings underline that ABX-induced microbiota reduction in 5xFAD mice is not sufficient to modulate Aβ uptake by microglia as observed in GF 5xFAD animals.

### Permanent absence of microbes alters microglial gene profile in GF 5xFAD mice

**Fig. 4:**
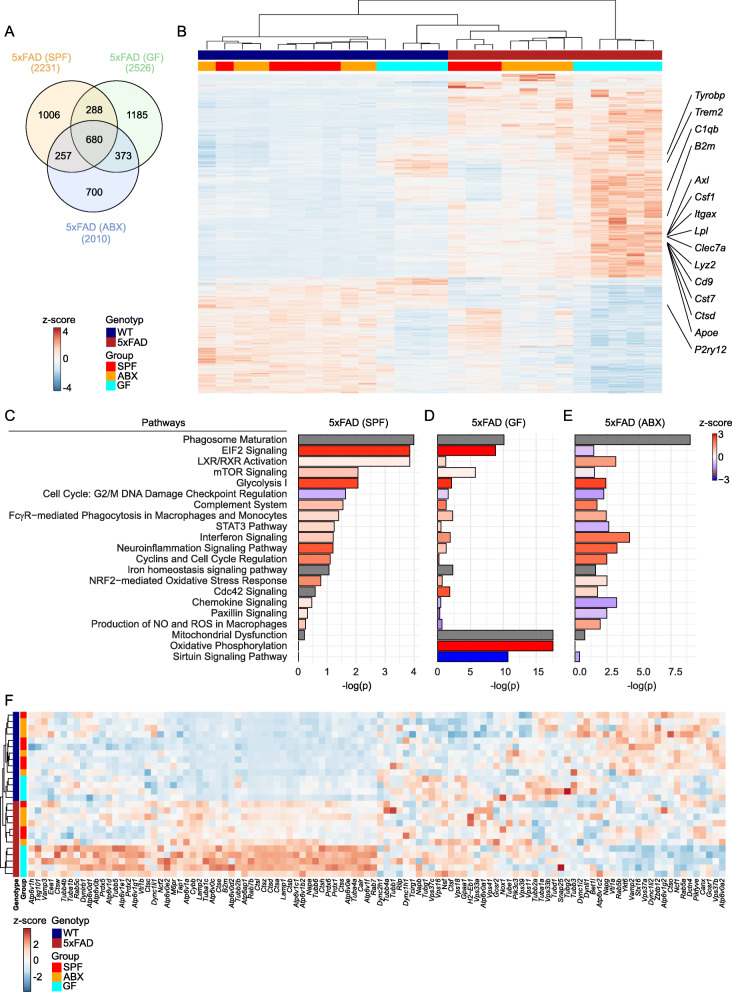
Altered microglial gene expression profiles in 5xFAD mice housed under GF conditions. (**a**) Venn diagram depicting the different regulated and overlapping genes between FACS-isolated hippocampal microglia of SPF, GF and ABX-treated 5xFAD animals compared to respective non-transgenic littermates of the same conditions (SPF/GF/ABX). (**b**) RNA-Seq analysis presenting mRNA expression profile of genes, whose expression was either induced or reduced with an adjusted *P* value < 0.0001 in hippocampal microglia of SPF, GF and ABX-treated transgenic 5xFAD animals and respective age-matched WT littermates. Representative genes are noted on the right. One column represents microglia from one individual mouse. Three to five mice were investigated per condition. Color code presents z-score (red: upregulated, blue: downregulated). Ingenuity pathway analysis (Qiagen) on differentially expressed genes in hippocampal microglia of SPF (**c**), GF (**d**) and ABX-treated (**e**) 5xFAD animals compared to respective WT littermates of the same housing/treatment conditions (SPF/GF/ABX) based on an RNA-sequencing analysis. Diagram depicts –log(p) value and predicted activation z-scores (red: increased activity, blue: reduced activity; grey: no predicted z-score available). (**f**) Heatmap of differently expressed genes attributed to the pathway ‘phagosome maturation’ of hippocampal microglia from SPF, GF and ABX-treated transgenic 5xFAD animals and respective age-matched WT littermates. Representative genes are noted on the bottom. One row represents microglia from one individual mouse. Three to five mice were investigated per condition. Color code presents z-score (red: upregulated, blue: downregulated)

To examine the effector functions of microglia in 5xFAD mice lacking microbes in more detail, we FACS-isolated hippocampal microglia from 4 months old SPF, GF and ABX 5xFAD mice and the respective age-matched WT controls and analyzed genome-wide mRNA expression profiles by RNA-sequencing (seq) (Fig. 4A & B). In general, 680 genes were similarly modulated under SPF, ABX and GF conditions in 5xFAD mice compared with non-transgenic WT controls (Fig. 4A, Additional file 8). Notably, microglia from SPF 5xFAD animals regulated 1006 individual genes, whereas microglia from GF 5xFAD mice displayed specific expression changes in 1185 genes and microglia in ABX-treated 5xFAD mice exhibited 700 uniquely transcribed gene transcripts (Fig. 4A). Statistical analysis of the most differently expressed genes across SPF and GF housing conditions and ABX treatment in WT and 5xFAD mice revealed 1945 significantly (adj. *P* < 0.0001) up- (red) and downregulated (blue) genes in microglia (Fig. 4B, Additional file 9). Among the differently expressed genes, we observed in microglia from GF 5xFAD mice upregulated expression of apolipoprotein E (*Apoe*) and *Trem2*, which are implicated in Aβ detection and clearance [44, 60]. Moreover, genes attributed to AD-associated microglial activation [23, 27] including *Axl*, *Cst7*, *Itgax* (encoding CD11c), *Cd9* or *Clec7a* showed an overall increased and *P2ry12* reduced expression in microglia from GF 5xFAD mice (Fig. 4B). Subsequent IPA pathway analysis of differentially expressed genes between microglia from WT and transgenic 5xFAD mice, housed under SPF or GF conditions or after ABX treatment, revealed strongly attenuated chemokine and mTOR signaling pathways in microglia from GF and ABX-treated 5xFAD mice, whereas neuroinflammation signaling pathways were robustly diminished in microglia from GF 5xFAD mice, leading to a strongly diminished repertoire of innate immune response (Fig. 4C – E, Additional files 10, 11, and 12). In contrast, microglia in ABX-treated 5xFAD mice displayed increased activation z-score of interferon signaling and Liver X Receptor (LXR) Retinoid X Receptor (RXR) activation, which belong to the type II family of nuclear receptors (NR). LXR and RXR signaling are involved in a wide range of Aβ-related effects and are also reported to promote anti-inflammatory effects in microglia [64]. Remarkably, we detected predominantly in microglia from GF 5xFAD mice robustly increased expression of genes attributed to oxidative phosphorylation and mitochondrial dysfunction, while the induction of genes linked to production of nitric oxide and reactive oxygen species were reduced concomitantly (Fig. 4D, Additional file 11). Further, we noticed an increased expression of genes implicated in phagocytosis, such as phagosome maturation and Cdc42 signaling [26], predominantly in microglia from GF 5xFAD mice (Fig. 4C – F, Additional files 10, 11, 12). In sum, the data is indicating an altered activation state of microglia in GF 5xFAD mice, while microglial phagocytosis is preferentially enhanced under GF conditions.

**Fig. 5:**
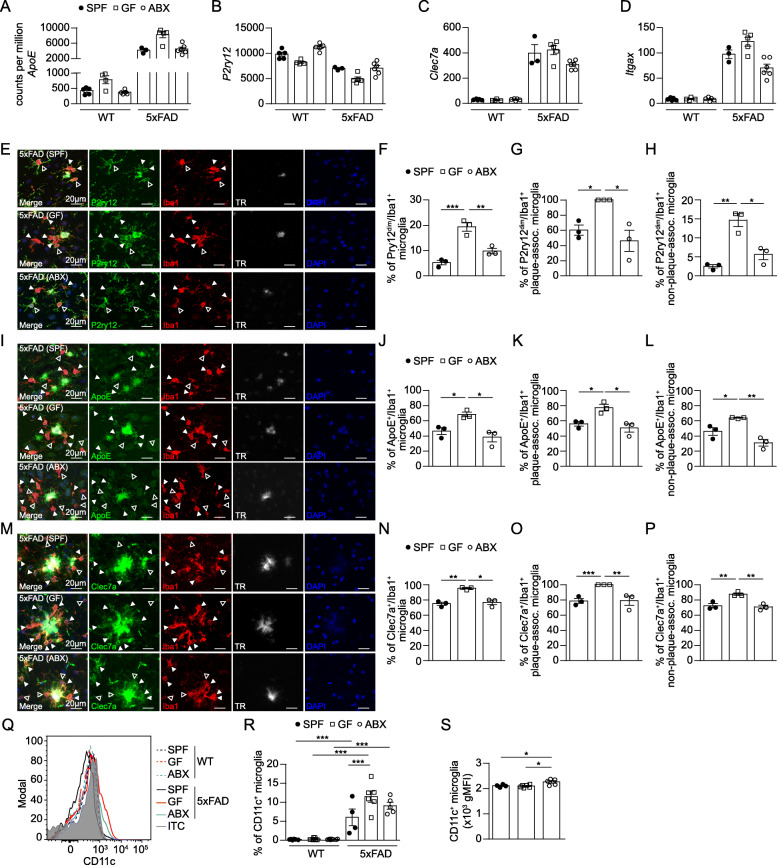
Altered expression of activation markers in hippocampal microglia from GF 5xFAD mice. Expression levels (counts per million) of (**a**) *ApoE*, (**b**) *P2ry12*, (**c**) *Clec7a* and (**d**) *Itgax* in hippocampal microglia from SPF, GF and ABX-treated 5xFAD and age-matched WT mice, based on RNA-seq data depicted in Fig. 4. Each symbol represents one mouse. Data are presented as mean ± s.e.m. (**e**) Representative immunofluorescence images of Iba1^+^ (red), P2ry12^+^ (green) microglia and TR^+^ (white) Aβ on parasagittal hippocampal sections from SPF, GF and ABX-treated 5xFAD mice. Nuclei were stained with DAPI (blue). Scale bar: 50 μm. White arrowheads indicate P2ry12^dim^/Iba1^+^ microglia and non-filled arrowheads show P2ry12^bright^/Iba1^+^ microglia. Quantification of the percentage of (**f**) total parenchymal P2ry12^dim^/Iba1^+^ microglia (**g**) TR^+^ plaque-associated P2ry12^dim^/Iba1^+^ microglia and (**h**) non-plaque-associated P2ry12^dim^/Iba1^+^ microglia in hippocampi of SPF, GF and ABX-treated 5xFAD mice. (**i**) Representative immunofluorescence images of Iba1^+^ (red) ApoE^+^ (green) microglia and TR^+^ (white) Aβ on parasagittal hippocampal sections from SPF, GF and ABX-treated 5xFAD mice. Nuclei were stained with DAPI (blue). Scale bar: 50 μm. White arrowheads indicate ApoE^+^/Iba1^+^ microglia and non-filled arrowheads show ApoE^−^/Iba1^+^ microglia. Quantification of the percentage of (**j**) total parenchymal ApoE^+^/Iba1^+^ microglia (**k**) TR^+^ plaque-associated ApoE^+^/Iba1^+^ microglia and (**l**) non-plaque-associated ApoE^+^/Iba1^+^ microglia in hippocampus from SPF, GF and ABX-treated 5xFAD mice. (**m**) Representative immunofluorescence images of Iba1^+^ (red) Clec7a^+^ microglia (green) and TR^+^ (white) on coronal hippocampal sections from SPF, GF and ABX-treated 5xFAD mice. Nuclei were stained with DAPI (blue). Scale bar: 50 μm. White arrowheads indicate Clec7a^+^/Iba1^+^ microglia and non-filled arrowheads show Clec7a^−^/Iba1^+^ microglia. Quantification of the percentage of (**n**) total parenchymal Clec7a^+^/Iba1^+^ microglia (**o**) TR^+^ plaque-associated Clec7a^+^/Iba1^+^ microglia and (**p**) non-plaque-associated Clec7a^+^/Iba1^+^ microglia in hippocampus of SPF, GF and ABX-treated 5xFAD mice. Each symbol represents one mouse. At least three slides were examined per individual mouse. Data are presented as mean ± s.e.m. Significant differences were determined by one-way ANOVA (*P < 0.05, **P < 0.01, ***P<0.001). Data are representative of two independent experiments. (**q**) Representative cytometric graph of CD11c^+^ labelled microglia from SPF (black line), GF (red line) and ABX-treated (blue line) 5xFAD mice and respective age-matched WT mice (dashed lines), compared to the isotype control (green line). In addition, quantifications of (**r**) percentages and (**s**) geometric mean fluorescence intensities (gMFI) of CD11c^+^ microglia cells are depicted. Each symbol represents one mouse. Data are presented as mean ± s.e.m. Significant differences were determined by two-way ANOVA followed by Bonferroni’s post-hoc comparison test or by one-way ANOVA followed by Tukey’s post-hoc comparison test (*P<0.05, ***P < 0.001). Data are representative of three independent experiments

Next, the differently expressed genes *ApoE* (Fig. 5A)*, P2ry12* (Fig. 5B)*, Clec7a* (Fig. 5C) and *Itgax* (Fig. 5D) were verified on protein level. We noticed an increased percentage of P2ry12^dim^/Iba1^+^ microglia in the hippocampus of GF 5xFAD mice (Fig. 5E & F), whereby TR^+^ plaque-associated microglia made up the majority of total P2ry12^dim^/Iba1^+^ cells (Fig. 5G & H). Furthermore, we confirmed increased *ApoE* and *Clec7a* expression in hippocampal microglia from GF 5xFAD mice as evident by increased percentages of ApoE^+^/Iba1^+^ (Fig. 5I – L) and Clec7a^+^/Iba1^+^ plaque-associated and non-plaque-associated microglia (Fig. 5M – P). Additionally, we observed elevated expression of CD11c^+^ hippocampal microglia from GF 5xFAD mice by flow cytometry (Fig. 5Q - S). Taken together, these findings demonstrate that the previously described AD-linked activation signature of microglia [23, 27] can be modulated by host microbiota.

### Microbiota-dependent enhanced microglial phagocytosis of Aβ debris is age-related

**Fig. 6:**
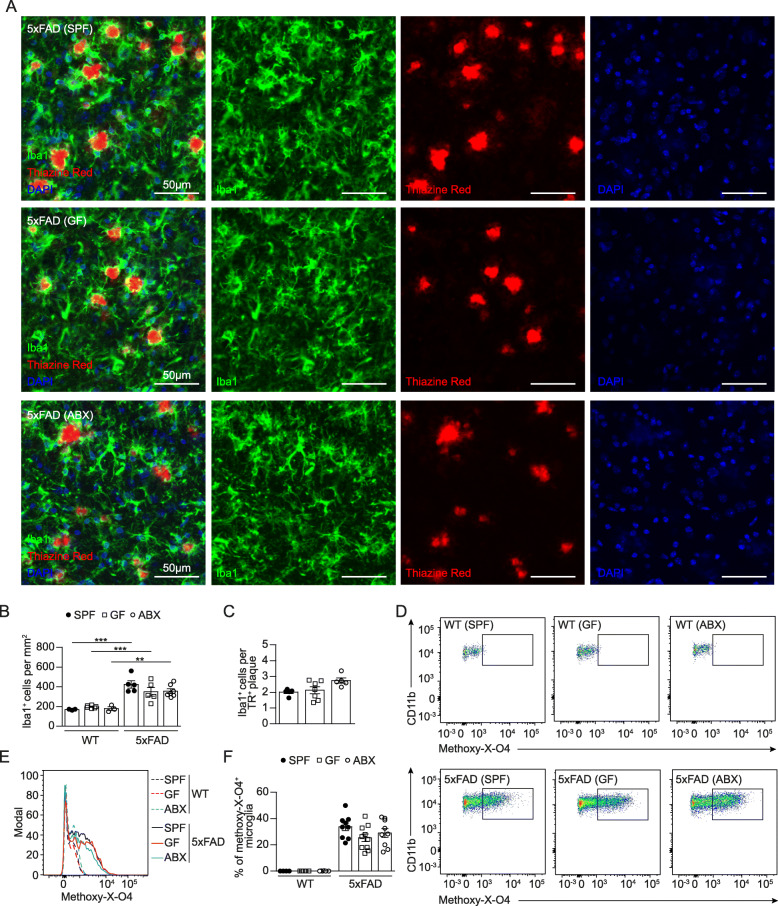
Equalized microglial Aβ phagocytosis in the hippocampus of aged 5xFAD mice. (**a**) Representative immunofluorescence images of TR (red) and Iba1 (green) on coronal hippocampal sections from 10 months old 5xFAD mice. Nuclei were stained with DAPI (blue). Scale bar: 50 μm. Quantification of (**b**) Iba1^+^ parenchymal in hippocampus of 5xFAD and age-matched WT mice. Quantification of (**c**) TR^+^ plaque-associated microglia in 5xFAD mice. Each symbol represents one mouse. Data are presented as mean ± s.e.m. Significant differences were determined by two-way ANOVA followed by Bonferroni post-hoc comparison test (**P < 0.01, ***P < 0.001). (**d**) Gating of CD11b^+^methoxy-XO4^+^microglia from SPF, GF and ABX-treated 5xFAD mice and age-matched WT controls. Representative flow cytometric dot plots are shown. (**e**) Representative cytometric graph of methoxy-X-O4^+^ labelled microglia from SPF (black line), GF (red line) and ABX-treated (blue line) 5xFAD mice and respective WT controls (dashed lines). (**f**) Quantification of percentages of methoxy-X-O4^+^ labelled microglia cells are depicted. Each symbol represents one mouse. Data are presented as mean ± s.e.m.: No significant differences were detected by two-way ANOVA followed by Bonferroni post-hoc comparison test. Data are representative of three independent experiments

Since it is well acknowledged that microglia become exhausted during chronic exposure to Aβ debris in mouse and human, we further examined whether microglial clearance capacity is sustained in later AD disease stage. First, we quantified Iba1^+^ parenchymal microglia numbers in the hippocampi of 10 months old SPF, GF and ABX 5xFAD mice and non-transgenic controls (Fig. 6A & B). In contrast to 4 months old animals, differences in microglial densities became indistinguishable in the 5xFAD mice at 10 months of age. In addition, the numbers of TR^+^ Aβ plaque-associated microglia were similar in all three experimental groups (Fig. 6C). In ex vivo flow cytometric analysis, the percentage of methoxy-X-O4^+^ microglia showed no differences (Fig. 6D – F, Suppl. Fig. 5B), thus demonstrating the age-dependency of microbiota-controlled microglial Aβ phagocytosis.

## Discussion

Recent studies uncovered an essential contribution of host microbiota during neurodegenerative diseases including AD, however, the cellular and functional mechanisms involved in the disease progression are not yet understood. Here, we examined the influence of distinct gut microbiota modulation strategies on microglia in the context of AD. Colonized 5xFAD mice, housed under SPF conditions, showed robust hippocampal Aβ pathology at early (4 months) and later disease stages (10 months), resulting thereby in disease stage-dependent neuronal loss and hippocampal-associated memory deficits. In contrast, constitutive (GF) or induced (ABX) depletion of the host’s bacteria significantly alleviated Aβ depositions and improved memory function. Importantly, constitutive and induced microbiota depletion strategies resulted in activation of different Aβ clearing mechanisms by the brain’s endogenous macrophages, the microglia. In GF 5xFAD mice microglial Aβ uptake was increased, while this was not the case upon ABX-treatment of 5xFAD mice.

In general, commensal bacteria are a well-known factor in maintaining the host’s physiology [41], including the innate and adaptive immune system in several tissues and compartments [16]. Moreover, alterations in the microbiota composition were linked to various human diseases, including several CNS disorders [7, 45, 55, 57, 58]. Their contribution to neurological health is studied extensively, however it is partially challenging to elucidate whether gut bacteria are a causative, propagative, or preventive factor during human health and disease. Nevertheless, growing evidence from preclinical studies suggests that gut bacteria are an essential factor contributing to CNS homeostasis and pathological conditions [6, 14, 20, 42, 50]. We previously identified that the commensal bacteria (presumably in the gut, due to their highest abundance) maintain microglial maturation and function in the CNS as evident by increased microglial numbers, highly branched arborization and altered gene expression patterns [12]. Interestingly, already during embryonic brain development, microglia features are essentially controlled by gut microbiota in a gender-dependent manner [54].

In accordance with our own and previous observations [18, 34] showing that host microbiota boost AD pathology, emerging evidence implies that gut microbes influence the pathogenesis of various neurodegenerative CNS diseases [4, 13, 43]. However, it remained largely unclear how this effect might be mediated. Harach and colleagues reported, for instance, less Iba1^+^ microglia accumulation in AD 3.5 and 8 months old mice under GF conditions [18]. In contrast, by using the more aggressive 5xFAD mouse model, we found in the hippocampus of GF housed transgenic mice an increased microglia density at 4 months of age. Of note, microglial numbers were equalized at later stages of the disease at 10 months of age. These data indicate certain limitations related to the different AD mouse models, as well as disease stage-dependent divergences. Despite the usage of constitutive gnotobiotic mouse models, which are highly artificial and untranslatable to the human situation, long-term manipulation of the gut microbiota with a cocktail of antibiotics was described to result in decreased Aβ burden in APP_SWE_/PS1_ΔE9_ AD model mice compared to non-treated controls [34]. In line, we observed diminished Aβ depositions in ABX-treated 5xFAD mice at both, early (4 months) and late (10 months) time points. This finding suggests that the disease pathology can be alleviated even at a progressed state, which could be useful for possible late therapeutic interventions. Notably, in ABX-treated 5xFAD mice microglial density turned out to be on same levels as observed in SPF housed transgenic controls, complementary to unchanged microglia abundance during homeostatic conditions in non-transgenic WT mice as described before [12, 35, 54]. Importantly, we determined that diminished Aβ burden in GF and ABX-treated 5xFAD mice resulted at later stages in reduced neuronal loss and in turn attenuated hippocampus-associated memory loss.

How does the microbiota shape AD progression? Generally, the equilibrium between Aβ production and clearance is regarded to be crucial for the Aβ burden in AD brain [22]. In addition, impaired Aβ removal rather than increased Aβ generation has been associated to the etiology of sporadic AD in humans [32]. The production of Aβ and APP processing was not altered in transgenic SPF, GF and ABX-treated 5xFAD mice. However, GF 5xFAD mice displayed an increased microglia accumulation close to Aβ plaques, containing more Aβ debris intracellularly compared to 4 months old SPF and ABX-treated 5xFAD mice, indicative of amplified phagocytic removal of Aβ. Consistent with these observations, several molecules regulating phagocytosis, such as *Trem2*, its adapter TYRO protein tyrosine kinase binding protein (*Tyrobp*), complement receptors (e.g. complement C1q subcomponent subunit B, *C1qb*) and *Apoe* were elevated in microglia from GF 5xFAD mice. For the lipoproteins, and in particular APOE, it is known that compact Aβ aggregates can be phagocytosed more efficiently by microglia [52, 61]. Remarkably, several genes (such as *Clec7a*, *P2ry12*, *Itgax* and others), that have been recently associated to Aβ activated microglia in AD mouse models [23, 27], as well as in human AD brains [56, 63], displayed altered expression in microglia from GF 5xFAD mice, indicating a certain microbiota-dependent influence on this microglial activation state. In addition, the microglial expression levels of genes attributed to neuroinflammation and chemokine signaling pathways were suppressed under GF conditions and partially reduced after ABX treatment. Despite the potential direct neurotoxic effects of such molecules, as it was proposed in the context of Parkinson’s diseases [43], cytokine expression is also implicated in affecting microglial phagocytosis [3]. Of note, the observed elevated uptake of Aβ depositions in GF 5xFAD mice was found to be equalized at later stages of the disease. In contrast to GF 5xFAD mice, acutely induced microbiota depletion by ABX did not increase microglial uptake of Aβ at early or late stages of the disease as compared to the SPF condition, suggesting other mechanisms of microbiota-driven Aβ clearance.

It was further reported that conventionally housed transgenic APPPS1 mice harbor alterations in gut microbiota composition, including increased abundance of e.g. *Rikenellaceae*, compared to the WT littermates. In the 5xFAD mouse model, slight changes in the composition of the fecal microbiota were also described compromising *Bacteroidetes* and *Firmicutes* [5]. However, in our 16S analysis of caecal contents we observed no major alterations (with a q-value < 0.05). In a rather small study, microbiota analysis of AD patients revealed declined microbial diversity as compared with control patients [58]. However, clinical implications of microbiota alterations need to be further clarified in future studies.

In conclusion, we found that host microbiota controlled microglia-mediated uptake of Aβ depositions in the 5xFAD mouse model of AD, whereas we observed different effects of constitutive (as in GF conditions) and induced microbiota modulation (by ABX) as well as disease stage-dependent mechanisms. RNA-seq analysis of FACS-purified hippocampal microglia uncovered distinct microbiota-dependent gene expression patterns including genes attributed to e.g. phagocytosis or complement signaling. Further, we found genes ascribed to AD-linked activation of microglia such as *Cst7*, *Clec7a*, *Apoe* or *Itgax* being expressed in a microbiota-dependent manner. These results provide deeper knowledge about the high plasticity of the gut-microglia connection and treatment of microglia-mediated CNS diseases.

## Supplementary information

**Additional file 1: Supplementary Fig. 1**: Determination of intestinal bacterial loads and microbiota composition analysis. **(A**) Photograph of caeca from 4- and 10- months old SPF, GF and ABX-treated mice with ruler for scaling and (**B**) relative caecal weight referred to the body weight, and (**C**) absolute body weight. Each symbol represents one mouse. Data are presented as mean ± s.e.m. Significant differences were determined by one-way ANOVA followed by Tukey’s post-hoc comparison test (***P < 0.001). Data are representative of four independent experiments. (**D**) Gating on DAPI^−^Syto9^+^ live bacteria of fecal samples from SPF, GF and ABX-treated mice. Representative flow cytometric dot plots are shown. (**E**) Percentages of live gram+ and gram- bacteria and (**F**) quantification of live bacteria per mg fecal sample are depicted. Each symbol represents one mouse. Data are presented as mean ± s.e.m. Significant differences were determined by one-way ANOVA followed by Tukey’s post-hoc comparison test (***P < 0.001). Data are representative of four independent experiments. (**G-J**) Microbial species richness (alpha diversity; Shannon and Simpson indices) in caecal contents from 4 months old SPF and ABX-treated 5xFAD mice and respective age-matched WT controls. (**K**) Microbial clustering is shown based on Bray-Curtis dissimilarity principal coordinate analysis (PCoA) metrics of caecal contents from 4 months old SPF and ABX-treated 5xFAD mice and respective age-matched WT controls. Ellipsoids represent a 95% confidence interval surrounding each group. Non-parametric analysis of variance (Adonis) was used to test significant difference between groups on PCoA plot; *p* < 0.001 for tested groups.

**Additional file 2: Supplementary Fig. 2.** Absence of host microbiota reduces hippocampal Aβ depositions in 5xFAD mice. (**A**) Representative immunofluorescence images of 6E10^+^ (red) compact and diffuse Aβ plaques in the hippocampus of 4 months old SPF, GF and ABX-treated 5xFAD mice**.** Nuclei were stained with DAPI (blue). Overview of hippocampus and magnification of subiculum (dashed line) are shown. Scale bars represent 300 μm (overview) and 50 μm (insert). (**B**) Quantification of the number of 6E10^+^ Aβ-plaques per mm^2^, (**C**) percentage of 6E10^+^ area and (**D**) average 6E10^+^ plaque size (μm^2^) in coronal hippocampal sections of SPF, GF and ABX-treated 5xFAD mice. Each symbol represents one mouse. Data are presented as mean ± s.e.m. Significant differences were determined by one-way ANOVA followed by Tukey’s post-hoc comparison test (***P < 0.001). Data are representative of four independent experiments. (**E**) Insoluble Aβ42/Aβ40 and (**F**) soluble Aβ42/Aβ40 ratio of hippocampal brain extracts from 4 months old SPF, GF and ABX-treated 5xFAD mice. ELISA for (**G**) insoluble Aβ42, (**H**) insoluble Aβ40, (**I**) soluble Aβ42, (**J**) soluble Aβ40, **(K)** ratio of insoluble Aβ42/ Aβ40 and ratio of soluble Aβ42/ Aβ40 of hippocampal brain extracts of 10 months old SPF, GF and ABX-treated 5xFAD mice. Each symbol represents one mouse. Data are presented as mean ± s.e.m. Significant differences were determined by one-way ANOVA followed by Tukey’s post-hoc comparison test (**P < 0.01, ***P < 0.001). Data are representative of two independent experiments. (**L**) Representative immunoblots of hippocampal brain homogenates of 10 months old SPF, GF and ABX-treated 5xFAD mice against human APP-FL, CTF-β, CTF-α, BACE1, ADAM10, PS1, PS2, PEN2, Nicastrin, and Aβ (6E10). β-Actin was used as loading control. Each lane represents one mouse. Quantification of (**M**) APP-FL, (**N**), CTF-β, (**O**) CTF-α, (**P**) BACE1, (**Q**) ADAM10, (**R**) PS1, (**S**) PS2, (**T**) PEN2, (**U**) Nicastrin, and (**V**) Aβ (6E10) protein levels normalized to β-Actin. Each symbol represents one mouse. Data are presented as mean ± s.e.m. Significant differences were determined by one-way ANOVA followed by Tukey’s post-hoc comparison test (*P < 0.05, **P < 0.01). Homogenates of three mice per group were used. (**W**) Representative immunofluorescence images of 6E10^+^ compact and diffuse Aβ plaques in the hippocampus of 10 months old 5xFAD mice. Nuclei were stained with DAPI (blue). Overview of hippocampus and magnification of subiculum (dashed line) are shown. Scale bars represent 300 μm (overview) and 50 μm (insert). (**X**) Quantification of the number of 6E10^+^ Aβ-plaques per area (mm^2^), (**Y**) percentage of 6E10^+^ area and (**Z**) average 6E10^+^ plaque size (μm^2^). Each symbol represents one mouse. Data are presented as mean ± s.e.m. Significant differences were determined by one-way ANOVA followed by Tukey’s post-hoc comparison test (*P < 0.05, ***P < 0.001). Data are representative of three independent experiments.

**Additional file 3: Supplementary Fig. 3.** Memory function in 4 months old SPF, GF and ABX-treated 5xFAD mice. (**A**) Ratio of right versus left arm entries in the T-maze spontaneous alternation test of 10 months old SPF and GF or (**B**) SPF and ABX-treated 5xFAD and age-matched WT mice. (**C-F**) T-maze of 4 months old SPF and GF mice or (**G-J**) SPF and ABX-treated 5xFAD, as well as respective age-matched WT mice. (**K-M**) Novel object recognition task of 4 months old SPF and GF 5xFAD, as well as age-matched WT mice or (**N-P**) SPF and ABX-treated 5xFAD, as well as WT mice. Each symbol represents one mouse. Data are presented as mean ± s.e.m. Significant differences were determined by two-way ANOVA followed by Bonferroni’s post-hoc comparison test (*P < 0.05, **P < 0.01, ***P < 0.001). Data are representative of three independent experiments. (**Q**) Representative immunofluorescence images of NeuN^+^ neurons (green) and TR^+^ (red) compact Aβ-plaques in the subiculum of the hippocampus of 4 months old SPF, GF and ABX-treated 5xFAD and respective age-matched WT mice**.** Nuclei were stained with DAPI (blue). Overview of hippocampus and magnification of subiculum (dashed line) are shown. Scale bars represent 200 μm (overview) and 50 μm (insert). (**R**) Quantification of the number of NeuN^+^ neurons per mm^2^ in the subiculum (Sub) of sagittal hippocampal sections from SPF, GF and ABX-treated 5xFAD and WT mice. Each symbol represents one mouse. Data are presented as mean ± s.e.m. No significant differences were determined by two-way ANOVA followed by Bonferroni’s post-hoc comparison test. Data are representative of two independent experiments.

**Additional file 4: Supplementary Fig. 4.** Microglial density in hippocampus of 4 months old 5xFAD mice. (**A**) Representative immunofluorescence images of Iba1^+^ (green) microglia on coronal hippocampal sections of 4 months old SPF, GF and ABX-treated WT mice. Nuclei were stained with DAPI (blue). Scale bar: 50 μm. (**B**) Representative immunofluorescence images of 6E10 (red) and Iba1 (green) on coronal hippocampal sections of 4 months old 5xFAD mice. Nuclei were stained with DAPI (blue). Scale bar: 50 μm. (**C**) Quantification of 6E10^+^ plaque-associated microglia. Each symbol represents one mouse. Data are represented as means ± s.e.m. Significant differences were determined by one-way ANOVA followed by Tukey’s post-hoc comparison test (**P < 0.01, ***P < 0.001). Data are representative of two independent experiments.

**Additional file 5: Supplementary Fig. 5.** Gating of hippocampal microglia in 5xFAD mice and non-transgenic controls. Gating of CD11b^+^CD45^low^ microglia from (A) 4 months old or (B) 10 months old SPF, GF and ABX-treated 5xFAD mice and age-matched WT controls. Representative dot plots are shown.

**Additional file 6.** Table 1. Maaslin Analysis Output (H2O Group for Genotype Comparison).

**Additional file 7.** Table 2. Maaslin Analysis Output (ABX Groups for Genotype Comparison).

**Additional file 8.** Table 3. Venn-diagram related genes.

**Additional file 9.** Table 4. List of DEG.

**Additional file 10.** Table 5. Pathway analysis SPF FAD vs WT.

**Additional file 11.** Table 6. Pathway analysis GF FAD vs WT.

**Additional file 12.** Table 7. Pathway analysis ABX FAD vs WT.

## Data Availability

All sequencing data (RNA-seq) are available at Gene Expression Omnibus (GEO: GSE154428).
