# Supplementary figures and images for: Different effects of constitutive and induced microbiota modulation on microglia in a mouse model of Alzheimer’s disease
Source: Acta Neuropathol Commun. 2020 Jul 29;8:119. doi: 10.1186/s40478-020-00988-5 (PMC7389451; doi:10.1186/s40478-020-00988-5)

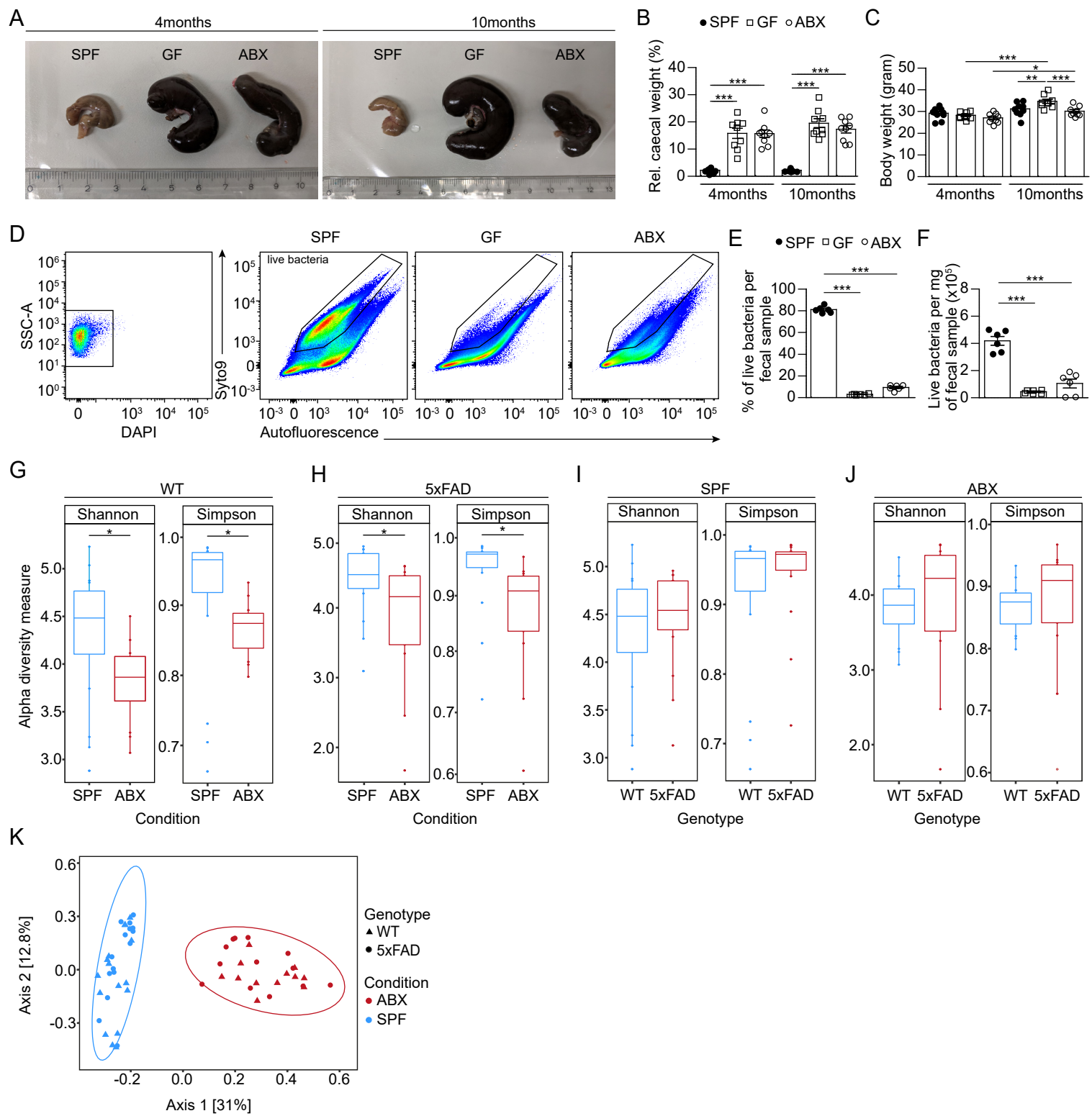

Supplement: Supplementary file 1 — Additional file 1: Supplementary Fig. 1: Determination of intestinal bacterial loads and microbiota composition analysis. (A) Photograph of caeca from 4- and 10- months old SPF, GF and ABX-treated mice with ruler for scaling and (B) relative caecal weight referred to the body weight, and (C) absolute body weight. Each symbol represents one mouse. Data are presented as mean ± s.e.m. Significant differences were determined by one-way ANOVA followed by Tukey’s post-hoc comparison test (***P < 0.001). Data are representative of four independent experiments. (D) Gating on DAPI−Syto9+ live bacteria of fecal samples from SPF, GF and ABX-treated mice. Representative flow cytometric dot plots are shown. (E) Percentages of live gram+ and gram- bacteria and (F) quantification of live bacteria per mg fecal sample are depicted. Each symbol represents one mouse. Data are presented as mean ± s.e.m. Significant differences were determined by one-way ANOVA followed by Tukey’s post-hoc comparison test (***P < 0.001). Data are representative of four independent experiments. (G-J) Microbial species richness (alpha diversity; Shannon and Simpson indices) in caecal contents from 4 months old SPF and ABX-treated 5xFAD mice and respective age-matched WT controls. (K) Microbial clustering is shown based on Bray-Curtis dissimilarity principal coordinate analysis (PCoA) metrics of caecal contents from 4 months old SPF and ABX-treated 5xFAD mice and respective age-matched WT controls. Ellipsoids represent a 95% confidence interval surrounding each group. Non-parametric analysis of variance (Adonis) was used to test significant difference between groups on PCoA plot; p < 0.001 for tested groups. [file 40478_2020_988_MOESM1_ESM.pdf]

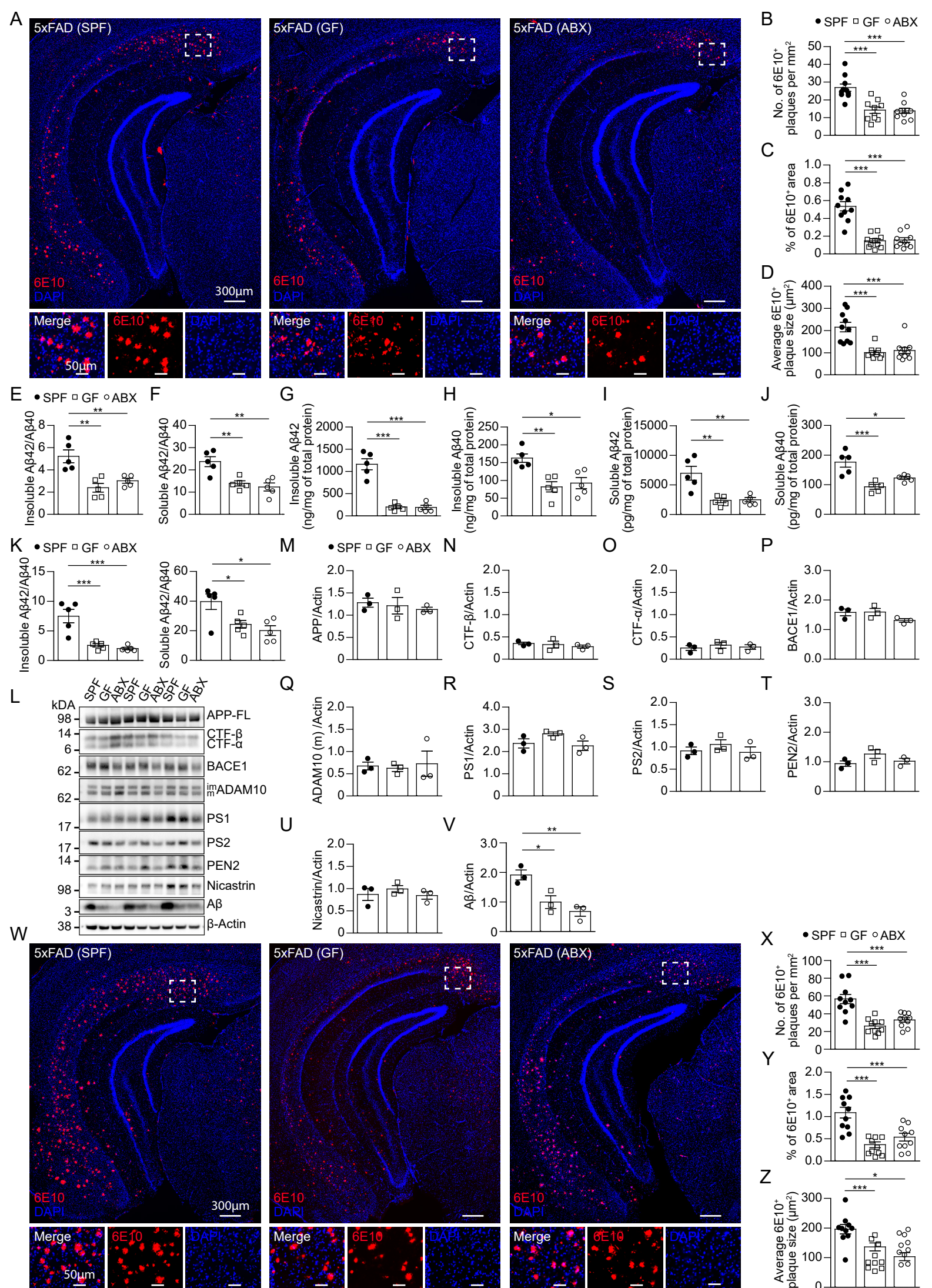

Suppl. Figure 2

Supplement: Supplementary file 2 — Additional file 2: Supplementary Fig. 2. Absence of host microbiota reduces hippocampal Aβ depositions in 5xFAD mice. (A) Representative immunofluorescence images of 6E10+ (red) compact and diffuse Aβ plaques in the hippocampus of 4 months old SPF, GF and ABX-treated 5xFAD mice. Nuclei were stained with DAPI (blue). Overview of hippocampus and magnification of subiculum (dashed line) are shown. Scale bars represent 300 μm (overview) and 50 μm (insert). (B) Quantification of the number of 6E10+ Aβ-plaques per mm2, (C) percentage of 6E10+ area and (D) average 6E10+ plaque size (μm2) in coronal hippocampal sections of SPF, GF and ABX-treated 5xFAD mice. Each symbol represents one mouse. Data are presented as mean ± s.e.m. Significant differences were determined by one-way ANOVA followed by Tukey’s post-hoc comparison test (***P < 0.001). Data are representative of four independent experiments. (E) Insoluble Aβ42/Aβ40 and (F) soluble Aβ42/Aβ40 ratio of hippocampal brain extracts from 4 months old SPF, GF and ABX-treated 5xFAD mice. ELISA for (G) insoluble Aβ42, (H) insoluble Aβ40, (I) soluble Aβ42, (J) soluble Aβ40, (K) ratio of insoluble Aβ42/ Aβ40 and ratio of soluble Aβ42/ Aβ40 of hippocampal brain extracts of 10 months old SPF, GF and ABX-treated 5xFAD mice. Each symbol represents one mouse. Data are presented as mean ± s.e.m. Significant differences were determined by one-way ANOVA followed by Tukey’s post-hoc comparison test (**P < 0.01, ***P < 0.001). Data are representative of two independent experiments. (L) Representative immunoblots of hippocampal brain homogenates of 10 months old SPF, GF and ABX-treated 5xFAD mice against human APP-FL, CTF-β, CTF-α, BACE1, ADAM10, PS1, PS2, PEN2, Nicastrin, and Aβ (6E10). β-Actin was used as loading control. Each lane represents one mouse. Quantification of (M) APP-FL, (N), CTF-β, (O) CTF-α, (P) BACE1, (Q) ADAM10, (R) PS1, (S) PS2, (T) PEN2, (U) Nicastrin, and (V) Aβ (6E10) protein levels normalized to β-Actin. Each symbol [file 40478_2020_988_MOESM2_ESM.pdf]

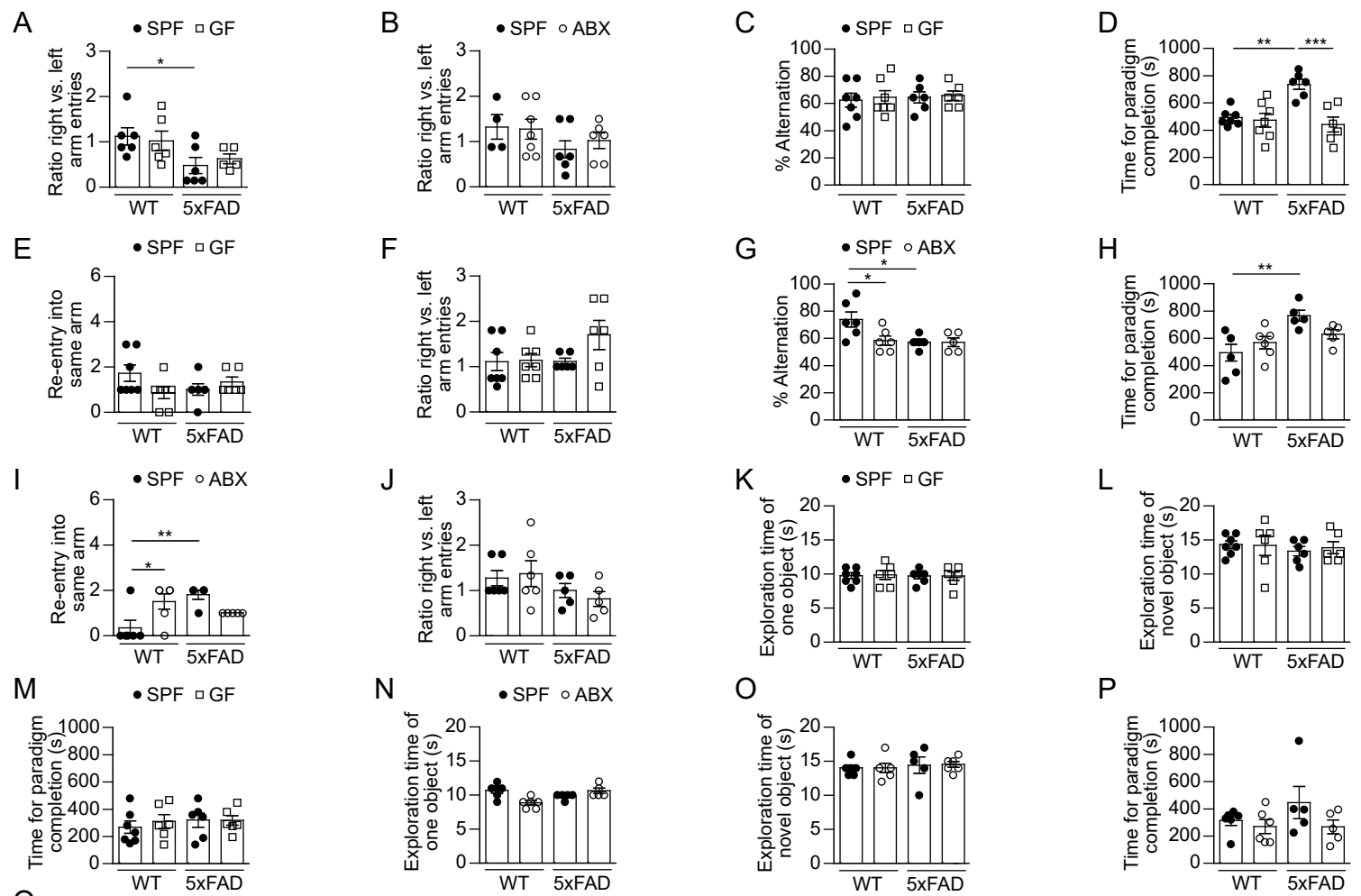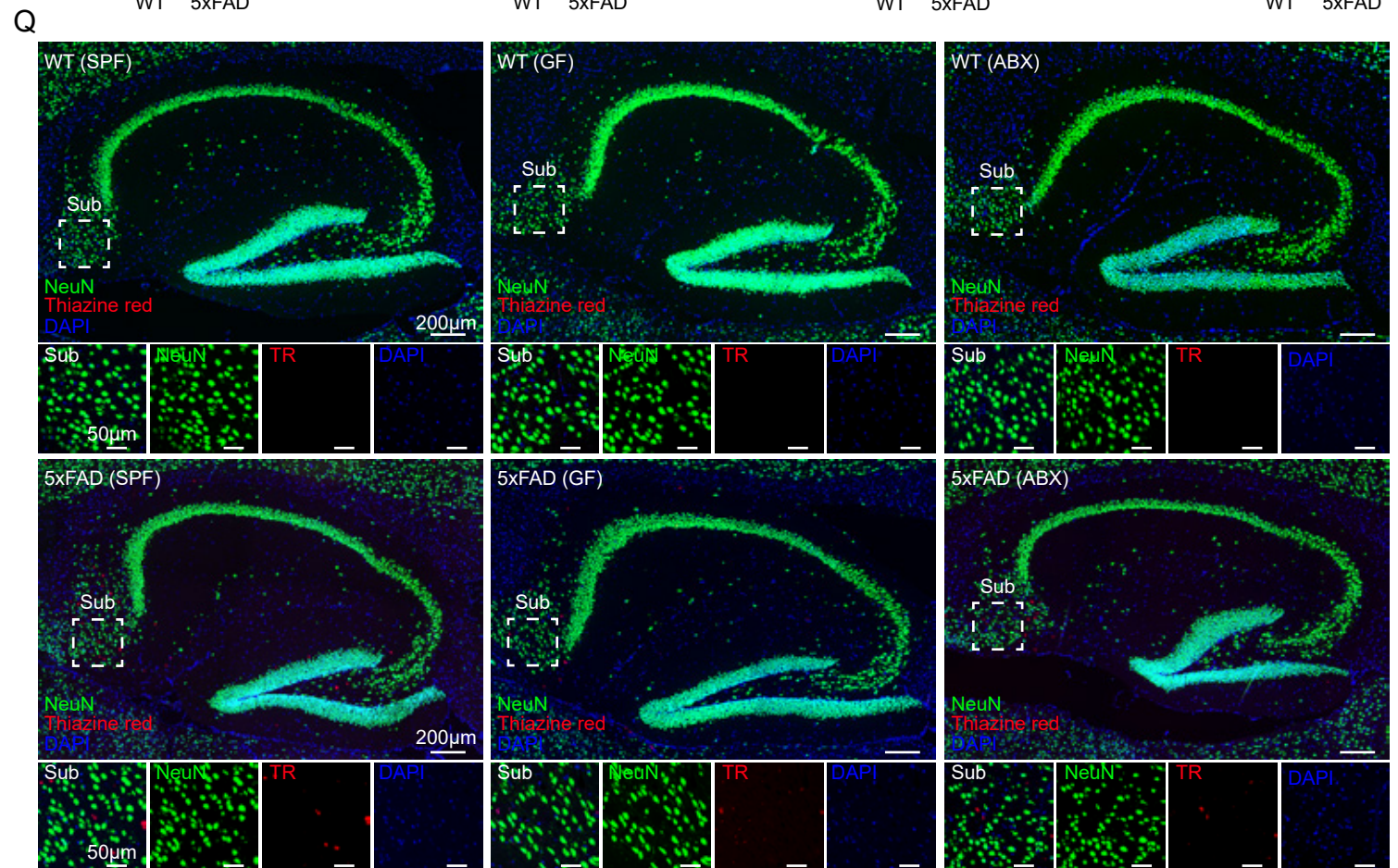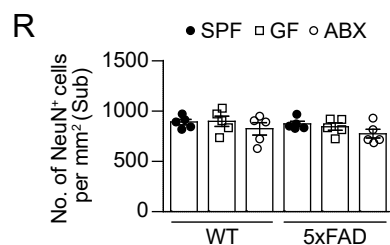

Suppl. Figure 3

Supplement: Supplementary file 3 — Additional file 3: Supplementary Fig. 3. Memory function in 4 months old SPF, GF and ABX-treated 5xFAD mice. (A) Ratio of right versus left arm entries in the T-maze spontaneous alternation test of 10 months old SPF and GF or (B) SPF and ABX-treated 5xFAD and age-matched WT mice. (C-F) T-maze of 4 months old SPF and GF mice or (G-J) SPF and ABX-treated 5xFAD, as well as respective age-matched WT mice. (K-M) Novel object recognition task of 4 months old SPF and GF 5xFAD, as well as age-matched WT mice or (N-P) SPF and ABX-treated 5xFAD, as well as WT mice. Each symbol represents one mouse. Data are presented as mean ± s.e.m. Significant differences were determined by two-way ANOVA followed by Bonferroni’s post-hoc comparison test (*P < 0.05, **P < 0.01, ***P < 0.001). Data are representative of three independent experiments. (Q) Representative immunofluorescence images of NeuN+ neurons (green) and TR+ (red) compact Aβ-plaques in the subiculum of the hippocampus of 4 months old SPF, GF and ABX-treated 5xFAD and respective age-matched WT mice. Nuclei were stained with DAPI (blue). Overview of hippocampus and magnification of subiculum (dashed line) are shown. Scale bars represent 200 μm (overview) and 50 μm (insert). (R) Quantification of the number of NeuN+ neurons per mm2 in the subiculum (Sub) of sagittal hippocampal sections from SPF, GF and ABX-treated 5xFAD and WT mice. Each symbol represents one mouse. Data are presented as mean ± s.e.m. No significant differences were determined by two-way ANOVA followed by Bonferroni’s post-hoc comparison test. Data are representative of two independent experiments. [file 40478_2020_988_MOESM3_ESM.pdf]

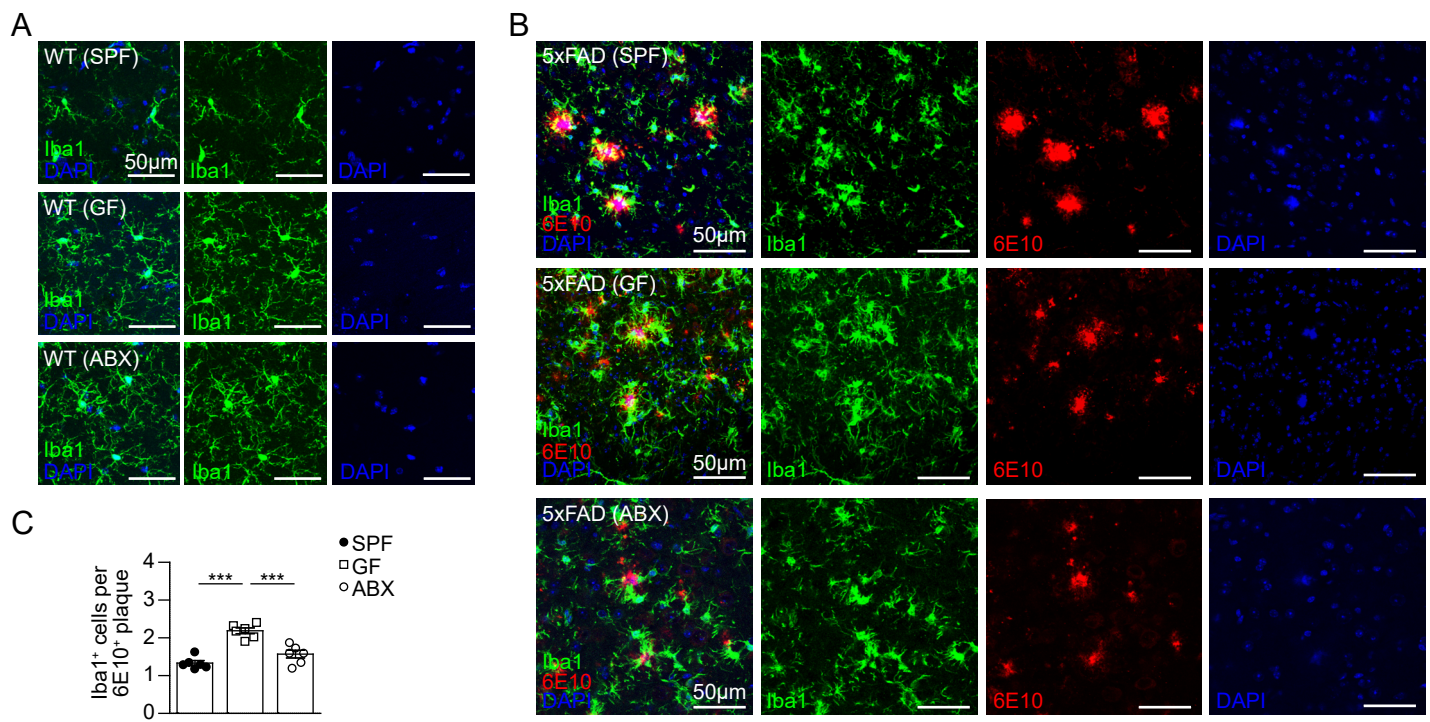

Suppl. Figure 4

Supplement: Supplementary file 4 — Additional file 4: Supplementary Fig. 4. Microglial density in hippocampus of 4 months old 5xFAD mice. (A) Representative immunofluorescence images of Iba1+ (green) microglia on coronal hippocampal sections of 4 months old SPF, GF and ABX-treated WT mice. Nuclei were stained with DAPI (blue). Scale bar: 50 μm. (B) Representative immunofluorescence images of 6E10 (red) and Iba1 (green) on coronal hippocampal sections of 4 months old 5xFAD mice. Nuclei were stained with DAPI (blue). Scale bar: 50 μm. (C) Quantification of 6E10+ plaque-associated microglia. Each symbol represents one mouse. Data are represented as means ± s.e.m. Significant differences were determined by one-way ANOVA followed by Tukey’s post-hoc comparison test (**P < 0.01, ***P < 0.001). Data are representative of two independent experiments. [file 40478_2020_988_MOESM4_ESM.pdf]

A

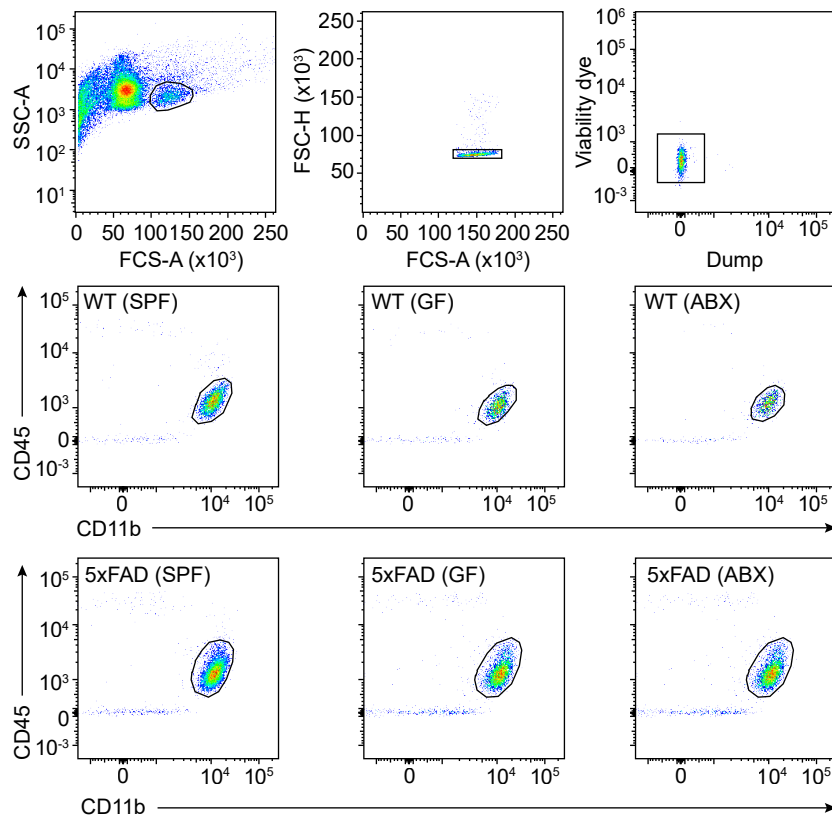

B

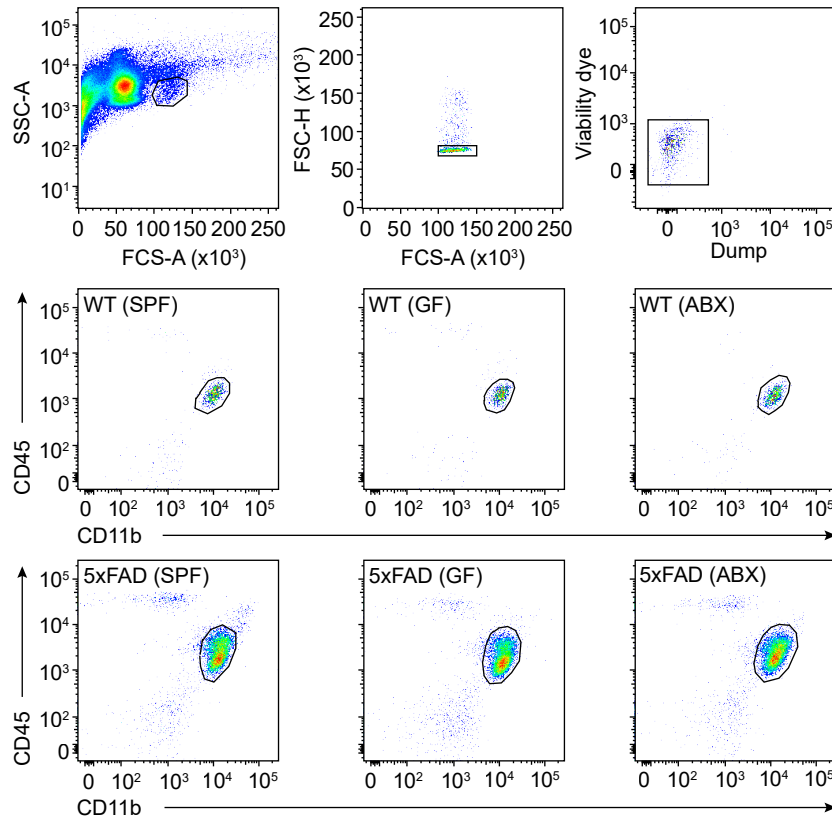

Supplement: Supplementary file 5 — Additional file 5: Supplementary Fig. 5. Gating of hippocampal microglia in 5xFAD mice and non-transgenic controls. Gating of CD11b+CD45low microglia from (A) 4 months old or (B) 10 months old SPF, GF and ABX-treated 5xFAD mice and age-matched WT controls. Representative dot plots are shown. [file 40478_2020_988_MOESM5_ESM.pdf]
